# Detection of common drug metabolites in urine using attenuated total reflectance-Fourier transform infrared spectroscopy (ATR-FTIR)

**DOI:** 10.1007/s12024-025-01017-4

**Published:** 2025-05-30

**Authors:** Yawen Yu, Tangdong Chen, Lijuan Yuan, Mao Sun, Yuanming Wu

**Affiliations:** 1 Department of Biochemistry and Molecular Biology, School of Basic Medicine, Shaanxi Provincial Key Laboratory of Clinic Genetics, Air Force Medical University, Xi’an, 710032 China; 2 Gastroenterology Department, Tongliang District Traditional Chinese Medicine Hospital, Chongqing, 402560 China; 3 Department of General Surgery, Tangdu Hospital, The Air Force Military Medical University, Xi’an, 710038 China; 4 Department of Clinical Laboratory, Tangdu Hospital, The Air Force Military Medical University, Xi’an, 710038 China

**Keywords:** Drug abuse, Metabolites, ATR-FTIR, Chemometrics, Forensic medicine

## Abstract

This study seeks to establish a rapid, non-invasive methodology for the detection of drug abuse through the identification of common urinary drug metabolites utilizing attenuated total reflectance Fourier transform infrared spectroscopy (ATR-FTIR). ATR-FTIR spectroscopy was employed to detect and differentiate metabolites of heroin (6-AM), ecstasy (MDA), and cocaine (BE) in urine samples across a range of concentrations. Advanced chemometric approaches, encompassing principal component analysis (PCA), partial least squares discriminant analysis (PLS-DA), and orthogonal partial least squares discriminant analysis (OPLS-DA), were applied to construct robust discriminative models. Spectral data from both the fingerprint region and the full spectral range were analyzed to maximize analytical precision. The proposed ATR-FTIR method demonstrated remarkable sensitivity, achieving detection of drug metabolites in urine at concentrations as low as 0.02 mg/mL without necessitating sample separation or extraction steps. The OPLS-DA model exhibited superior discriminative performance, effectively distinguishing all three metabolites in both calibration and validation sets. These findings underscore the potential of integrating ATR-FTIR with chemometrics for the development of a rapid and reliable drug screening tool. This pilot investigation demonstrates that the integration of ATR-FTIR spectroscopy with chemometric analysis represents a highly promising strategy for the detection of urinary drug metabolites, circumventing the need for complex sample pretreatment procedures. This approach offers a novel, efficient, and non-invasive solution for the rapid identification of drug abuse, with substantial implications for forensic medicine and public health surveillance.

**Supplementary Information:**

The online version contains supplementary material available at 10.1007/s12024-025-01017-4.

## Introduction

The issue of drug abuse has persistently remained a global concern. Despite concerted international efforts, including those by China, to combat illicit narcotics, the current landscape of global drug production, trafficking, and misuse continues to be alarmingly severe. Narcotics sources and types have diversified, the demographic of drug abusers has expanded to include younger populations, and the societal ramifications have grown increasingly profound, compounding the complexities of the international drug control scenario [1, 2]. While numerous novel psychoactive substances (NPS) continue to emerge, heroin retains its position as the most prevalent drug of abuse in China. By the end of 2023, registered heroin abusers in China totaled 305,000, constituting 34% of the nation’s 896,000 documented drug users [3]. Concurrently, methamphetamine seizures reached unprecedented levels in 2021, underscoring its status as the world’s most illicitly synthesized drug. Furthermore, the global supply and demand dynamics for cocaine have exhibited persistent escalation [2].

Urine serves as the biological matrix of choice for drug screening due to its non-invasive collection and extended detection window. Contemporary illicit drug testing methodologies predominantly rely on mass spectrometry (MS), the gold standard for drug analysis, often integrated with chromatographic systems such as high-performance liquid chromatography (HPLC) and high-resolution gas chromatography (HRGC) [4]. These techniques primarily target parent drugs and their metabolites, offering exceptional accuracy and sensitivity for trace substance detection. However, inherent limitations still exist, as equipment is often expensive and cumbersome to operate, and often requires professional personnel to operate [5, 6]. Immunoassays, while widely employed for preliminary drug screening, suffer from compromised specificity and sensitivity, potentially yielding false-positive or -negative results that undermine diagnostic reliability [7].

Fourier transform infrared spectroscopy (FTIR) is a qualitative and quantitative analysis technique based on molecular vibration theory [8–10]. The infrared spectrum is measured by measuring the interferogram and Fourier change of the interferogram. According to the different characteristics of the spectrogram, the functional group of the unknown substance, the chemical structure, and the chemical reaction process, etc., can be obtained [10]. In particular, the attenuated total reflection (ATR) mode of FTIR is a widely used technique in infrared spectroscopy analysis. The core of ATR-FTIR lies in the interaction between infrared light and the sample at the interface of a high-refractive-index crystal, typically made of diamond, zinc selenide (ZnSe), or germanium (Ge) [8]. When infrared light is directed into the ATR crystal at an angle greater than the critical angle, it undergoes total internal reflection. During this process, an evanescent wave is generated at the crystal-sample interface. The evanescent wave penetrates a short distance into the sample, where it interacts with the molecular vibrations of the sample [11]. Molecular functional groups exhibit distinct vibrational modes in the mid-infrared region, primarily categorized into stretching vibrations and bending vibrations, as detailed in the supplementary information (Fig S1) [12].

The absorption of specific infrared wavelengths by sample molecules, corresponding to their characteristic vibrational frequencies, produces a unique spectral “fingerprint.” This attenuated radiation is subsequently detected by the spectrometer, yielding a spectral profile that decodes molecular composition and conformational details. The technique’s rapid analysis time and operational simplicity render it advantageous for high-throughput screening [13, 14]. Prior investigations have demonstrated ATR-FTIR’s applicability for detecting illicit substances in biological fluids including saliva and urine [15, 16].

Chemometrics is the application of mathematical and statistical analyses to chemical data, enabling the extraction of meaningful information from multivariate data sets [17]. It is a highly promising, advantageous, and productive analytical method that currently plays an indispensable role in the investigation of practical analysis problems. It can optimize the measurement process of relevant experiments, effectively extract experimental data, establish a reasonable mathematical model, and obtain valuable information needed by researchers [18]. Chemometrics is widely used in the field of forensic medicine. Furthermore, the ATR-FTIR technique has been used and combined with chemometrics methods to identify the narcotics in a range of concentrations with different cuttings [4]. In this study, ATR-FTIR combined with chemometrics was used to detect urine samples with the addition of different concentrations of drug metabolites (6-acetylmorphine (6-AM), benzoylecgonine (BE) and 3,4-methylenedioxyamphetamine (MDA)) in order to apply it to the field of forensic medicine for the rapid identification of drug abusers.

## Materials and methods

### Experimental samples

The standard reference materials for 6-AM, BE and MDA used in this study were all purchased from the FENGE Standards (Beijing, China). All urine samples were from the sample bank of provincial key laboratory of clinical genetics (Shaanxi, China). Rigorous exclusion criteria were applied to ensure sample integrity and study validity: volunteers with pre-existing medical conditions, recent (within 30 days) drug use, history of blood transfusion, or surgical interventions were excluded. These measures effectively minimized potential confounders, ensuring that collected urine samples accurately represented the study objectives without interference from extraneous medical variables, recent pharmacokinetics, or iatrogenic factors. All studies were approved by the ethics approval from the Air Force Medical University (Approval No. KY20241765-F-1).

### Sample preparation

2.2.1 A total of 210 urine samples were combined in equal aliquots, and 15 mL was taken as the working solution (Blank urine sample). A total of 50 additional urine samples were combined in equal aliquots, and 10 mL was taken as the working solution to form an independent validation set. The concentration of each drug standard solution was 1 mg/mL. Based on the different drugs added (6-AM, BE, and MDA), the urine samples with the added standards were divided into three groups, and five different concentrations of samples were prepared for each group. A 1 mL volumetric flask was used for the preparation of different concentrations of drugs, and the urine working solution was used for volume determination. Specific concentration formulation information is provided in supplementary information Table S1.

#### Method validation

①Limit of Detection (LOD): A concentration gradient was set for each sample group to determine the methodological limit of detection (LOD) for 6-AM, BE, and MDA.

②Precision: Prepared samples were stored at 4 °C. Within-run precision was assessed at intervals of 2, 4, and 6 h within one day, and between-run precision was assessed at intervals of 1, 3, and 5 days.

### ATR-FTIR spectra acquisition and data preprocessing

All spectral measurements were performed using a Nicolet iS50 FTIR spectrometer (Thermo Fisher Scientific, Waltham, MA, USA) equipped with a diamond ATR sampling module. Spectral processing was executed through OMNIC v9.2 software (Thermo Nicolet Analytical Instruments, WI, USA). Acquisition parameters were set as follows: 32 scans at 4 cm⁻¹ resolution across the 400–4000 cm⁻¹ range. Prior to each measurement cycle, the ATR crystal was cleaned with anhydrous ethanol, air-dried, and baseline-corrected using atmospheric background subtraction. Sample preparation involved pipetting 2 µL aliquots onto the ATR crystal, followed by 4-minute forced-air drying to ensure complete solvent evaporation. Spectral stability was verified through moisture interference checks. Triplicate measurements were performed for each sample, with nine spectra averaged per specimen to enhance signal-to-noise ratio and minimize experimental variance. Automatic baseline correction was applied using the instrument’s acquisition software.

### Chemometrics algorithms and software

#### Unsupervised method

Unsupervised method provides exploratory insights into multivariate data structures by revealing latent relationships among variables and observations [19, 20]. Principal component analysis (PCA) was selected as the primary unsupervised technique for dimensionality reduction, facilitating the identification of spectral discriminators between drug-containing urine samples and their concentration-dependent variations. PCA transforms correlated spectral variables into orthogonal principal components (PCs), capturing maximal variance with reduced dimensionality while preserving critical discriminative features [21].

#### Supervised method

Partial least squares discriminant analysis (PLS-DA) is a supervised classificatory technique that integrates dimensionality reduction with discriminant modeling, particularly suited for high-dimensional spectral datasets. By projecting both predictor and response variables into a latent space, PLS-DA generates interpretable weight-loading relationships that elucidate discriminative patterns [22]. The PLS-DA version with orthogonal signal correction (OPLS-DA) was also used in this study. It makes the corresponding predictive scores and loading vectors less subject to orthogonal variation, thereby enhancing model robustness against experimental biases and biological variability [23, 24].

#### Model implementation

Discriminative models were constructed using PCA, PLS-DA, and OPLS-DA algorithms. Statistical analyses were performed in SPSS v26.0 (IBM, USA) and SIMCA v14.1 (Umetrics, Sweden). Data visualization was accomplished using Origin v2021 (OriginLab, USA).

## Results

### Comparison of average ATR‑FTIR spectra from blank urine samples and 6-AM, BE, and MDA standards

**Fig. 1:**
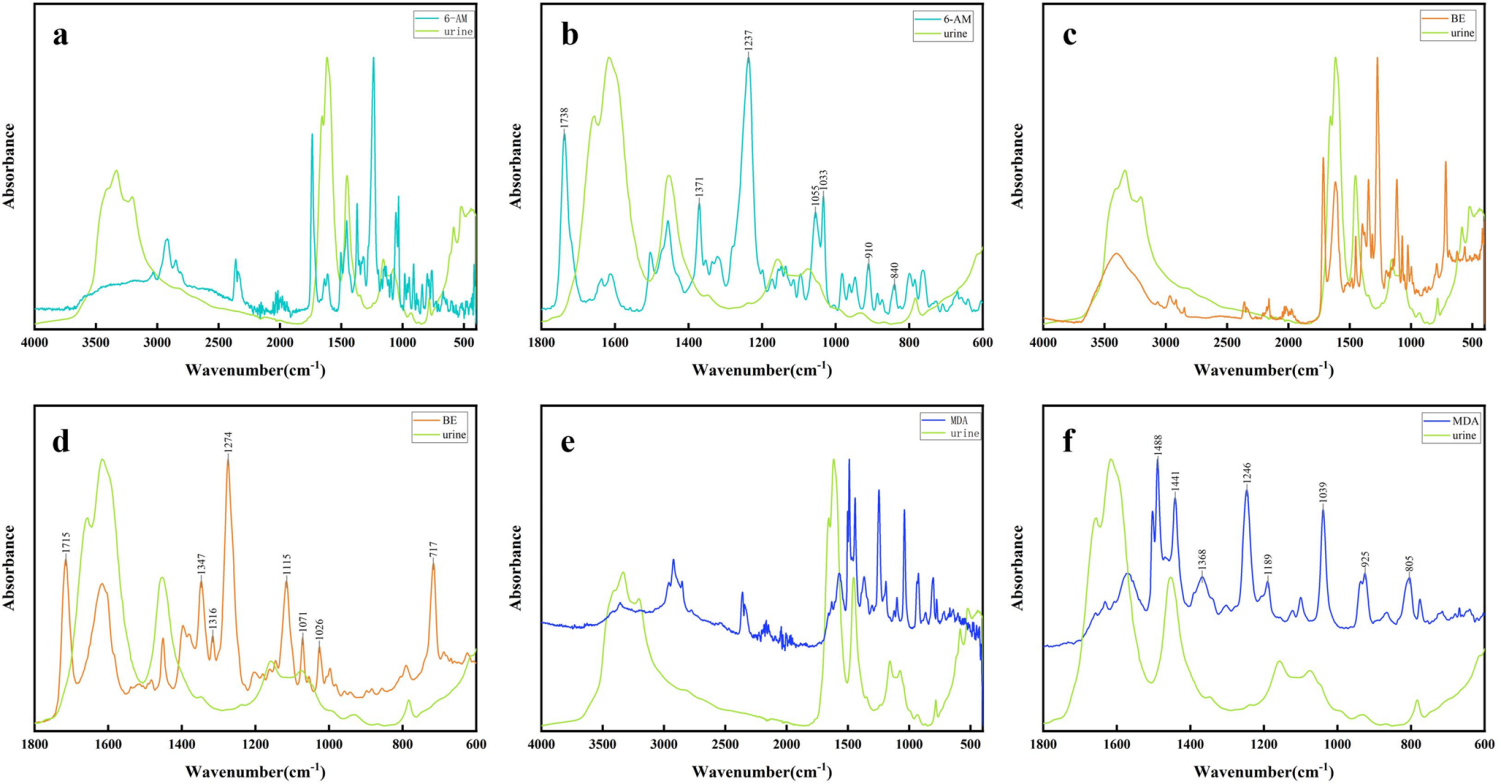
Infrared spectra of blank urine and standard samples of 6-AM, BE, and MDA: (**a**-**b**) Comparison of ATR-FTIR spectra of the standard of 6-AM and blank urine. (**c**-**d**) Comparison of ATR-FTIR spectra of the standard of BE and blank urine. (**e**-**f**) Comparison of ATR-FTIR spectra of the standard of MDA and blank urine

Figure 1 shows the infrared spectra of blank urine and standard samples of 6-AM, BE, and MDA, including both the full spectral region (Fig. 1a, c and e) and the fingerprint region (Fig. 1b, d and f). The main absorption peaks of standard samples of 6-AM, BE, and MDA in the fingerprint regions are marked in Fig. 1. In Fig. 1b, the main absorption peaks were at 1738 cm^− 1^, 1371 cm^− 1^, 1237 cm^− 1^, 1055 cm^− 1^, 1033 cm^− 1^, 910 cm^− 1^, and 840 cm^− 1^. Among them, the peak at 1738 cm^− 1^ was due to C = O stretching vibration, the peak at 1371 cm^− 1^ was the -CH_3_ bending vibration, the peak at 1237 cm^− 1^, 1055 cm^− 1^ and 1033 cm^− 1^ were the C-O stretching vibration, the peak at 910 cm^− 1^ and 840 cm^− 1^ were due to C-H out-of-plane bending vibration [25, 26]. In Fig. 1d, the main absorption peaks were at 1715 cm^− 1^, 1347 cm^− 1^, 1316 cm^− 1^, 1274 cm^− 1^, 1115 cm^− 1^, 1071 cm^− 1^, 1026 cm^− 1^, and 717 cm^− 1^. Among them, the peak at 1715 cm^− 1^ was due to C = O stretching vibration, the peak at 1347 cm^− 1^ and 1316 cm^− 1^ were the -CH_3_ bending vibration, the peak at 1274 cm^− 1^ was due to C-N stretching vibration, the peak at 1115 cm^− 1^, 1071 cm^− 1^ and 1026 cm^− 1^ were due to C-O stretching vibration, and the peak at 717 cm^− 1^ was the C-H out-of-plane bending vibration [27–29]. In Fig. 1f, the main absorption peaks were observed around 1488 cm^− 1^, 1441 cm^− 1^, 1368 cm^− 1^, 1246 cm^− 1^, 1189 cm^− 1^, 1039 cm^− 1^, 925 cm^− 1^, and 805 cm^− 1^. Among them, the peak at 1488 cm^− 1^ and 1441 cm^− 1^ were due to C = C stretching vibration, the peak at 1368 was -CH_3_ bending vibration, the peak at 1246 cm^− 1^, 1189 cm^− 1^ and 1039 cm^− 1^ were attributed to C-O bending vibration, the peak at 925 cm^− 1^ and 805 cm^− 1^ were due to C-H out-of-plane bending vibration [30]. The characteristic absorption peaks and their corresponding attributions for 6-AM, BE, and MDA are summarized in Supplementary Table S2.

### Method validation of average ATR‑FTIR spectra for urine samples with added standards

**Fig. 2:**
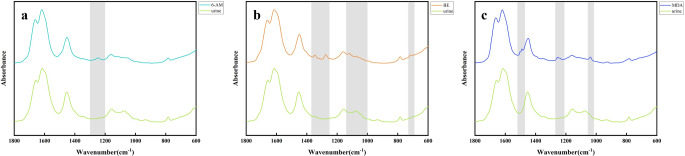
Infrared spectra of blank urine and urine with added standards of 6-AM, BE, and MDA: (**a**) Comparison of infrared spectra of blank urine (green) and urine added with 6-AM (0.02 mg/mL; turquoise). (**b**) Comparison of infrared spectra of blank urine (green) and urine added with BE (0.02 mg/mL; orange). (**c**) Comparison of infrared spectra of blank urine (green) and urine added with MDA (0.02 mg/mL; blue)

The detection limits of the three metabolites were evaluated via infrared spectroscopy. Figure 2 illustrates the minimum detectable concentrations of 6-AM, BE, and MDA in spiked urine samples using this approach. The gray-shaded regions highlight metabolite-specific absorption features discernible at these concentrations. Detection thresholds were established by spectral comparison between spiked urine and blank controls. The lowest concentration at which characteristic absorptions remained discernible was 0.02 mg/mL.

Compared to the blank urine sample, only one characteristic absorption peak of 6-AM is observed at 1200 cm^− 1^ to 1300 cm^− 1^, consistent with the characteristic peak at 1237 cm^− 1^ mentioned above (Fig. 2a). The characteristic absorption peaks found in BE at 1365–1200 cm^− 1^ and 740 − 700 cm^− 1^ correspond to the characteristic peaks at 1316 cm^− 1^, 1274 cm^− 1^and 717 cm^− 1^ mentioned above (Fig. 2b). MDA exhibited characteristic peaks not present in urine at 1520–1475 cm^− 1^, 1280–1220 cm^− 1^, and 1060–1020 cm^− 1^, which correspond to the characteristic peaks mentioned above: 1488 cm^− 1^, 1246 cm^− 1^, and 1039 cm^− 1^ (Fig. 2c).

### Identification of mixed complex metabolites in urine by chemometrics

#### Complex metabolites identification model by PCA

We explored the feasibility of ATR-FTIR coupled with chemometrics for urinary drug metabolite detection using PCA on spiked urine samples (Group A, B, M). However, neither full spectral analysis (4000–400 cm⁻¹) nor fingerprint region analysis (1800–600 cm⁻¹) achieved satisfactory class separation. While score plots (Fig. S3a, S3c) suggested general clustering by group, distinct segregation was lacking. Loading plot (Fig. S3) showed the result of PC-1 loading. The loading plot of PC-1 using spectral fingerprint regions (Fig. S3b) accounted for 44.5% of the variation in this model, while the loading plot of PC-1 using full spectral regions (Fig. S3d) accounted for 29.1%.

#### Complex metabolites identification model by PLS-DA

Given PCA’s limited classification performance, a supervised PLS-DA approach was adopted. Models were trained on 2/3 of samples (10 per metabolite) and validated on the remaining 1/3 (5 per metabolite).

**Fig. 3:**
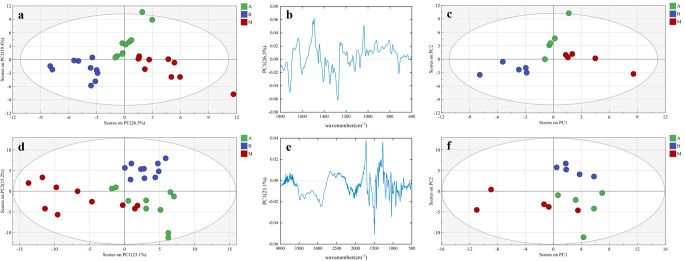
PLS-DA score plots and loading plots: (**a**) PLS-DA model score plot using fingerprint regions in the calibration set. (**b**) PC-1 loading plot of the PLS-DA model using fingerprint regions. (**c**) PLS-DA model score plot using fingerprint regions in the validation set. (**d**) PLS-DA model score plot using full spectral regions in the calibration set. (**e**) PC-1 loading plot of the PLS-DA model using full spectral regions. (**f**) PLS-DA model score plot using full spectral regions in the validation set

PLS-DA was used as a supervised method to detect and identify different metabolites in urine samples with added standards. The spectral fingerprint regions (Fig. 3a and c) and full spectral regions (Fig. 3d and f) of the urine samples with added standards were selected for establishing the classification model. Using spectral fingerprint regions, seven PCs were chosen to build a PLS-DA model (Table S5; Fig. S4a). Group B (B) could be effectively distinguished from group A (A) and group M (M). However, group M could not be completely distinguished from Group A in the calibration set. The loading plot of PC-1 (Fig. 3b) corresponds to 26.5% of the variation in this model. The permutation test (200 times) was performed as internal validation of the model (Fig. S4b). External validation of the model was performed in the validation set (Fig. 3c). ROC analysis (Fig. S5) was performed to evaluate the model, suggesting that this model is accurate and stable. The full spectral regions of urine were also selected for conducting the PLS-DA model (Fig. 3d and f). Eight PCs were opted for modeling after variable optimization (Table S6; Fig. S4c). In the model, group A, group M, and group B were broadly distinguished in the calibration set, especially group A. The loading plot of PC-1 (Fig. 3b) corresponds to 23.1% of the variation in this model. Internal validation of the model was conducted using a permutation test (200 iterations) (Fig. S4d), and external validation was performed on the validation set (Fig. 3f). External validation in an independent set also showed good clustering performance (Fig. S8a).

#### Complex metabolites identification model by OPLS-DA

**Fig. 4:**
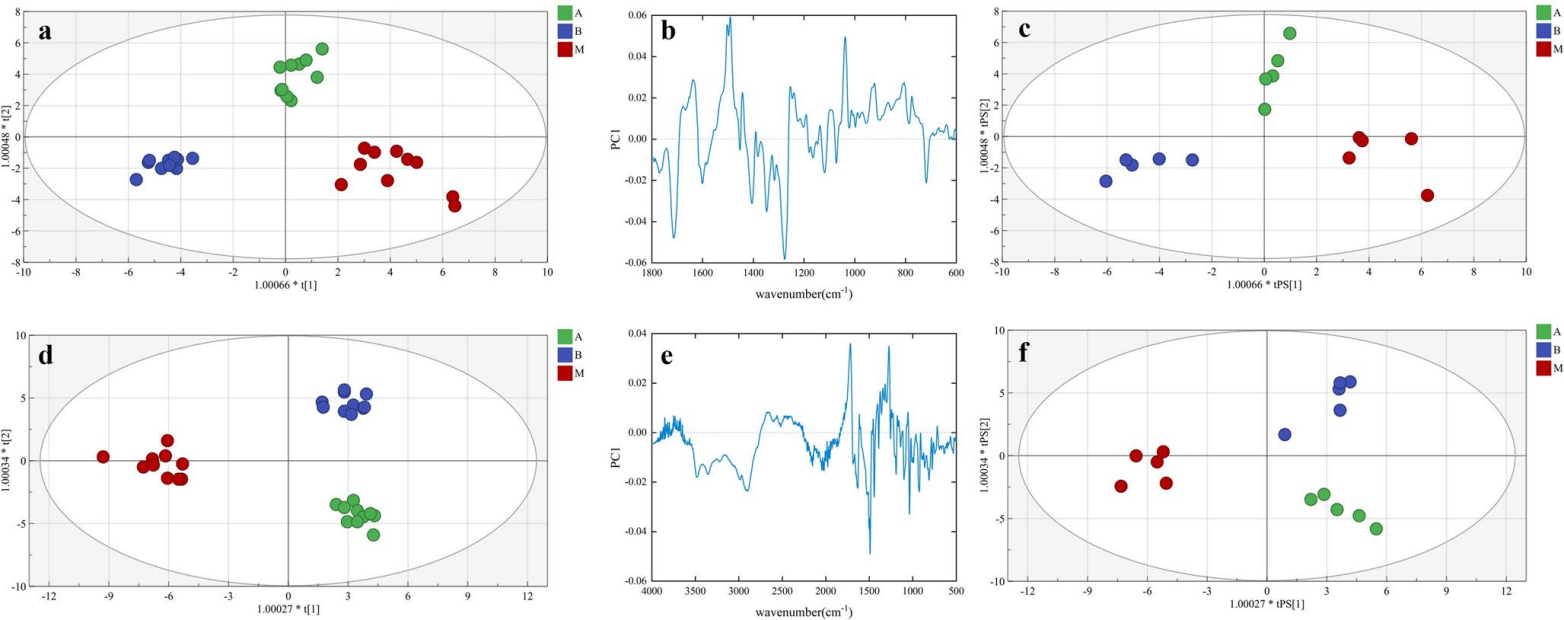
OPLS-DA score plots and loading plots: (**a**) OPLS-DA model score plot using fingerprint regions in the calibration set. (**b**) Loading plot of the OPLS-DA model using fingerprint regions. (**c**) OPLS-DA model score plot using fingerprint regions in the validation set. (**d**) OPLS-DA model score plot using full spectral regions in the calibration set. (**e**) Loading plot of the OPLS-DA model using full spectral regions. (**f**) OPLS-DA model score plot using full spectral regions in the validation set

Furthermore, OPLS-DA was used to build classification models for identification of additives in the urine. Spectral fingerprint regions of urine were selected for the first time (Fig. 4a and c). Two PCs were chosen to build the OPLS-DA model after variable optimization (Table S7; Fig. S6a). In the calibration set, group A, group M and group B could be distinguished completely and efficiently (Fig. 4a). Figure 4b shows the PC-1 loading plot of the model. Internal validation was conducted using a permutation test (200 iterations) (Fig. S6b), and external validation was performed on the validation set (Fig. 4c). ROC analysis was performed to evaluate the OPLS-DA model suggesting that this model is stable and accurate (Fig. S7). The full spectral regions were also selected for classification modeling (Fig. 4d and f). Two PCs were the optimal choice for modeling after variable optimization (Table S8; Fig. S6c). Internal validation was conducted using a permutation test (200 iterations) (Fig. S6d). In the calibration set, groups A, M, and B were also distinguished completely and efficiently (Fig. 4d). Figure 4e presents the PC-1 loading plot of the model. External validation of the model was also conducted on the validation set (Fig. 4f). External validation in an independent set also showed good clustering performance (Fig. S8b).

## Discussion

Here, we initially conducted a comparative analysis of the average ATR-FTIR spectra derived from blank urine samples and the 6-AM, BE, and MDA standards. This comparative spectral examination was undertaken to delineate their respective characteristic absorption peaks and corresponding specific functional groups. Given the study’s focus on the identification and differentiation of drug metabolites in urine, further examination of the parent drugs was deemed beyond the scope of this investigation. It is important to highlight that codeine metabolism can also yield morphine, which is not exclusive to heroin. Consequently, the presence of morphine alone cannot serve as definitive confirmation of heroin use. To circumnavigate this ambiguity, the more specific metabolite 6-AM was selected as the target analyte for heroin identification. Notably, these functional groups are frequently encountered in genuine metabolic processes, such as those occurring in urine. However, urine itself exhibits inherent absorption bands that may potentially interfere with the detection of target substances, either enhancing or obscuring their characteristic peaks.

Next, we employed infrared spectroscopy to evaluate the limit of detection (LOD) for these three metabolites. The findings from both within-run and between-run precision assessments demonstrated remarkable stability, underscoring the reliability and reproducibility of our experimental methodology. Furthermore, infrared spectroscopy offers distinct advantages over mass spectrometry in terms of convenience and speed for metabolite identification, while also surpassing the sensitivity of rapid test strips [31–33]. Collectively, these attributes render this approach highly generalizable and promising for future practical applications in forensic toxicology.

ATR-FTIR was subsequently utilized to detect metabolites of heroin, ecstasy, and cocaine in urine, across varying concentrations. Qualitative analysis of these metabolites was executed using both supervised and unsupervised algorithms, culminating in the establishment of classification models for the three substances. These models yielded satisfactory classification outcomes, confirming the discriminative power of the technique even in the presence of urinary matrix interference.

As is well-established, algorithms such as PCA, PLS-DA, and OPLS-DA, which were employed in this study, belong to the category of linear dimensionality reduction methods. While effective for linearly correlated data, real-world datasets often exhibit complex, nonlinear structures that may elude accurate representation by linear approaches. Given the escalating diversity of drugs and the growing number of polydrug users, future research could explore nonlinear dimensionality reduction techniques, such as diffusion map embedding (DME), or hybrid methods integrating multiple dimensionality reduction strategies. Such advancements could lead to the development of more robust and accurate discrimination models for distinguishing metabolites of multiple drugs in complex matrices.

To the best of our knowledge, this study represents the inaugural application of ATR-FTIR spectroscopy in conjunction with chemometrics for the detection of common drug metabolites in urine. FTIR spectroscopy is a mature technology with established utility in forensic science, as evidenced by prior literature [13, 15, 28, 34]. However, within the domain of drug identification, FTIR has predominantly been applied to the qualitative or quantitative analysis of seized drugs or simulated adulterants [4, 16, 27, 35]. A distinguishing feature of our work is the emphasis on metabolite identification in urine, which is a more clinically relevant matrix than seized drug materials. This focus necessitated the selection of 6-AM as a heroin biomarker, due to the aforementioned challenges associated with morphine’s non-specificity.

Despite these advancements, several limitations warrant acknowledgment. Firstly, the positive urine samples utilized in this study were artificially generated, owing to the inherent difficulties in obtaining authentic positive samples from real-world scenarios. Consequently, validation against genuine population samples remains an unfulfilled requirement. Secondly, while the technique demonstrated feasibility for rapid detection in urine, the current LOD of 0.02 mg/mL for metabolites is higher than concentrations typically encountered in clinical or forensic settings. This sensitivity threshold is notably inferior to established forensic methods like mass spectrometry, which operates at the nanogram level. However, there exist viable avenues for enhancing sensitivity, such as the incorporation of quantum cascade lasers (QCLs) as light sources. QCLs provide highly concentrated photon flux, augmenting target molecule absorption signals, and their narrow linewidth facilitates selective excitation of molecular peaks, thereby minimizing spectral interference from background absorbents like water [36]. Finally, the present study focused on single-metabolite detection, whereas real-world scenarios often involve the presence of multiple metabolites, including primary and secondary biotransformation products, alongside parent drugs. To enhance the practical utility of this method, future iterations should incorporate comprehensive testing for parent drugs and their metabolic byproducts.

## Conclusion

In this study, attenuated total reflection Fourier transform infrared spectroscopy (ATR-FTIR) was employed to detect metabolites of heroin, ecstasy, and cocaine in urine matrix at varying concentrations. By integrating ATR-FTIR with advanced chemometric tools, we developed robust classification models utilizing both supervised and unsupervised algorithms to qualitatively distinguish these drug metabolites. Notably, these models demonstrated exceptional discriminatory power, even in the presence of complex urinary matrix interferences.

A pivotal advantage of this methodology lies in its sample preparation simplicity—the proposed approach enables direct analysis of human urine without conventional pretreatment steps, or with minimal processing requirements. Remarkably, as little as 2 µL of urine was sufficient for accurate analysis, representing a significant reduction in sample volume compared to conventional liquid chromatography-tandem mass spectrometry (LC/MS-MS) techniques that typically require orders of magnitude larger volumes.

Preliminary exploration of the limit of detection (LOD) for urinary drug metabolites using ATR-FTIR spectroscopy was also conducted, yielding promising results. This technical advancement holds substantial promise for rapid field-based identification of drug abuse, particularly in forensic toxicology settings where speed and efficiency are critical. The findings presented herein not only validate the feasibility of ATR-FTIR as a portable and cost-effective screening tool but also underscore its potential to revolutionize on-site drug testing protocols.

## Key points

1. An objective and rapid method for the identification of drug abuse was presented.
2. First detection and distinction of 6-AM, MDA, BE in urine using ATR-FTIR technology.
3. ATR-FTIR combined with chemometrics showed potential roles for forensic identification.

## Electronic supplementary material

Below is the link to the electronic supplementary material.

Supplementary Material 1

## Data Availability

The datasets used and analyzed during the current study are presented in the figures,and they are available from the corresponding authors upon reasonable request.

## References

[1] Sun H, Bao Y, Zhou S, et al. The new pattern of drug abuse in China. Curr Opin Psychiatry. 2014;27(4):251–5. 10.1097/YCO.0000000000000073. 24840156 10.1097/YCO.0000000000000073

[2] World Drug Report. United Nations Office on Drugs and Crime. 2023. https://www.unodc.org

[3] China drug Situation Report. Office of the National Narcotics Control Commission of China. 2023. http://www.nncc626.com

[4] Williams SF, Stokes R, Tang PL, et al. Detection & identification of hazar-dous narcotics and new psychoactive substances using fourier transform infrared spectroscopy (FTIR). Anal Methods. 2023;15(26):3225–32. 10.1039/d3ay00766a. 37341678 10.1039/d3ay00766a

[5] Salerno TMG, Donato P, Frison G, et al. Gas Chromatography-Fourier transform infrared spectroscopy for unambiguous determination of illicit drugs: A proof of concept. Front Chem. 2020;8:624. 10.3389/fchem.2020.00624. 32850646 10.3389/fchem.2020.00624 PMC7396574

[6] Abiedalla Y, Almalki AJ, DeRuiter J, et al. GC-MS and GC-IR analysis of methylenedioxyphenylalkylamine analogues of the psychoactive 25X-NBOMe drugs. Forensic Chem. 2021;23:100314. 10.1016/j.forc.2021.100314.

[7] Liu L, Wheeler SE, Venkataramanan R, et al. Newly emerging drugs of abuse and their detection methods. Am J Clin Pathol. 2018;149(2):105–16. 10.1093/ajcp/aqx138. 29385414 10.1093/ajcp/aqx138

[8] Beć KB, Grabska J, Huck CW. Biomolecular and bioanalytical applications of infrared spectroscopy - A review. Anal Chim Acta. 2020;1133:150–77. 10.1016/j.aca.2020.04.015. 32993867 10.1016/j.aca.2020.04.015

[9] Berthomieu C, Hienerwadel R. Fourier transform infrared (FTIR) spectroscopy. Photosynth Res. 2009;101(2–3):157–70. 10.1007/s11120-009-9439-x. 19513810 10.1007/s11120-009-9439-x

[10] Chen T, Sun M, Li B, et al. Identifying hypothermia death in a mouse model by ATR-FTIR. Int J Legal Med. 2024;138(3):1179–86. 10.1007/s00414-023-03156-1. 38191742 10.1007/s00414-023-03156-1

[11] Gauglitz G, Moore DS. Handbook of spectroscopy: second, enlarged edition. Wiley; 2014. pp. 39–70. 10.1002/9783527654703.

[12] Wen S. Fourier transform infrared spectroscopy analysis. Chemical Indust-ry; 2010.

[13] Wei C, Wang J. A rapid and nondestructive approach for forensic identification of car bumper splinters using attenuated total reflectance fourier transfor-m infrared spectroscopy and chemometrics. J Forensic Sci. 2021;66(2):583–93. 10.1111/1556-4029.14606. 33113238 10.1111/1556-4029.14606

[14] He X, Wang J. Rapid and nondestructive forensic identification of tire particles by attenuated total Reflectance-Fourier transform infrared Spectroscop-y and chemometrics. Anal Lett. 2020;53(5):714–34. 10.1080/00032719.2019.1668947.

[15] Algethami FK, Eid SM, Kelani KM, et al. Chemical fingerprinting and qua-ntitative monitoring of the doping drugs bambuterol and Terbutaline in human urine samples using ATR-FTIR coupled with a PLSR chemometric tool. RSC Adv. 2020;10(12):7146–54. 10.1039/c9ra10033d. 35493915 10.1039/c9ra10033d PMC9049731

[16] Hans KMC, Müller S, Sigrist MW. Infrared attenuated total reflection (IR-ATR) spectroscopy for detecting drugs in human saliva. Drug Test Anal. 2012;4(6):420–9. 10.1002/dta.346. 22113850 10.1002/dta.346

[17] Weber A, Hoplight B, Ogilvie R, et al. Innovative vibrational Spectroscop-y research for forensic application. Anal Chem. 2023;95(1):167–205. 10.1021/acs.analchem.2c05094. 36625116 10.1021/acs.analchem.2c05094

[18] He X, Wang J, You X, et al. Classification of heroin, methamphetamine, ketamine and their additives by attenuated total reflection-Fourier transform infrared spectroscopy and chemometrics. Spectrochimica Acta Mol Biomol Spectrosc. 2020;241:118665. 10.1016/j.saa.2020.118665. 10.1016/j.saa.2020.118665 32683249

[19] Callao MP, Ruisánchez I. An overview of multivariate qualitative methods for food fraud detection. Food Control. 2018;86:283–93. 10.1016/j.foodcont.2017.11.034.

[20] Dhaulaniya AS, Balan B, Yadav A, et al. Development of an FTIR based chemometric model for the qualitative and quantitative evaluation of cane sugar as an added sugar adulterant in Apple fruit juices. Food Addit Contam Part Chem Anal Control Expo Risk Assess. 2020;37(4):539–51. 10.1080/19440049.2020.1718774. 10.1080/19440049.2020.1718774 32023186

[21] Zha S, Wei X, Fang R, et al. Estimation of the age of human semen stains by attenuated total reflection fourier transform infrared spectroscopy: a preliminary study. Forensic Sci Res. 2020;5(2):119–25. 10.1080/20961790.2019.1642567. 32939428 10.1080/20961790.2019.1642567 PMC7476623

[22] Fordellone M, Bellincontro A, Mencarelli F. Partial least squares discriminant analysis: A dimensionality reduction method to classify hyperspectral data. Stat Appl. 2019;31:181–200. 10.26398/IJAS.0031-010.

[23] Boccard J, Rutledge DN. A consensus orthogonal partial least squares discriminant analysis (OPLS-DA) strategy for multiblock omics data fusion. Anal Chim Acta. 2013;769:30–9. 10.1016/j.aca.2013.01.022. 23498118 10.1016/j.aca.2013.01.022

[24] De Mateus Pereira N, Hunter Machado B, Koche A, et al. Detectio-n of metabolic syndrome with ATR-FTIR spectroscopy and chemometrics in bl-ood plasma. Spectrochim Acta Mol Biomol Spectrosc. 2023;288:122135. 10.1016/j.saa.2022.122135. 10.1016/j.saa.2022.122135 36442341

[25] Stevanović NR, Jovanović M, Marini F, et al. Chemometric approach to a rapid attenuated total reflection fourier transform infrared analysis of Com-plex Heroin-Based mixtures. Appl Spectrosc. 2021;75(5):545–55. 10.1177/0003702820969715. 33052052 10.1177/0003702820969715

[26] Teoh WK, Mohamed Sadiq NS, Saisahas K, et al. Detection and discrimination of sedative-hypnotics in spiked beverage dry residues using attenuated total reflectance‐Fourier transform infrared (ATR‐FTIR) spectroscopy combined with chemometrics. J Forensic Sci. 2023;68(1):75–85. 10.1111/1556-4029.15156. 36273275 10.1111/1556-4029.15156

[27] Rodrigues NVS, Cardoso EM, Andrade MVO, et al. Analysis of seized cocaine samples by using chemometric methods and FTIR spectroscopy. J Braz Chem Soc. 2013;24(3):507–17. 10.5935/0103-5053.20130066.

[28] Materazzi S, Gregori A, Ripani L, et al. Cocaine profiling: implementation of a predictive model by ATR-FTIR coupled with chemometrics in forensic chemistry. Talanta. 2017;166:328–35. 10.1016/j.talanta.2017.01.045. 28213242 10.1016/j.talanta.2017.01.045

[29] Marcelo MCA, Mariotti KC, Ferrão MF, et al. Profiling cocaine by ATR-FTIR. Forensic Sci Int. 2015;246:65–71. 10.1016/j.forsciint.2014.11.011. 25460107 10.1016/j.forsciint.2014.11.011

[30] Tsujikawa K, Kuwayama K, Miyaguchi H, et al. Development of an on-site screening system for amphetamine-type stimulant tablets with a portable attenuated total reflection fourier transform infrared spectrometer. Anal Chim Acta. 2008;608(1):95–103. 10.1016/j.aca.2007.12.002. 18206999 10.1016/j.aca.2007.12.002

[31] Concheiro M, Simões SMDS, Quintela Ó, et al. Fast LC–MS/MS method f-or the determination of amphetamine, methamphetamine, MDA, MDMA, MDE-A, MBDB and PMA in urine. Forensic Sci Int. 2007;171(1):44–51. 10.1016/j.forsciint.2006.10.004. 17097252 10.1016/j.forsciint.2006.10.004

[32] Marchei E, Colone P, Nastasi GG, et al. On-site screening and GC–MS analysis of cocaine and heroin metabolites in body-packers urine. J Pharm Biomed Anal. 2008;48(2):383–7. 10.1016/j.jpba.2007.11.025. 18164159 10.1016/j.jpba.2007.11.025

[33] Fiorentin TR, D’Avila FB, Comiran E, et al. Simultaneous determination of cocaine/crack and its metabolites in oral fluid, urine and plasma by liquid chromatography-mass spectrometry and its application in drug users. J Pharmacol Toxicol Methods. 2017;86:60–6. 10.1016/j.vascn.2017.04.003. 28395991 10.1016/j.vascn.2017.04.003

[34] Wei X, Yu K, Wu D, et al. Species identification of semen stains by ATR-FTIR spectroscopy. Int J Legal Med. 2021;135(1):73–80. 10.1007/s00414-020-02367-0. 32647962 10.1007/s00414-020-02367-0

[35] Pereira LSA, Lisboa FLC, Coelho Neto J, et al. Screening method for rapi-d classification of psychoactive substances in illicit tablets using mid infrared spectroscopy and PLS-DA. Forensic Sci Int. 2018;288:227–35. 10.1016/j.forsciint.2018.05.001. 29777946 10.1016/j.forsciint.2018.05.001

[36] Schwaighofer A, Brandstetter M, Lendl B. Quantum cascade lasers (QCLs) in biomedical spectroscopy. Chem Soc Rev. 2017;46:5903–24. 10.1039/c7cs00403f. 28816307 10.1039/c7cs00403f

