# Supplementary material for: Detection of common drug metabolites in urine using attenuated total reflectance-Fourier transform infrared spectroscopy (ATR-FTIR)
Source: Forensic Sci Med Pathol. 2025 May 30;21(4):1617–25. doi: 10.1007/s12024-025-01017-4 (PMC12799672; doi:10.1007/s12024-025-01017-4)
Supplement: Supplementary file 1 — Supplementary Material 1 [file 12024_2025_1017_MOESM1_ESM.docx]

**Supplementary Information**

**Detection of Common Drug Metabolites in Urine Using Attenuated Total Reflectance-Fourier Transform Infrared Spectroscopy (ATR-FTIR)**

**Table S1** Ratio table of different sample concentrations

| Add substances | Groups | Sample number | Concentration  (mg/mL) |
| --- | --- | --- | --- |
| 6-AM | A | A-1 | 0.02 |
|  |  | A-2 | 0.04 |
|  |  | A-3 | 0.05 |
|  |  | A-4 | 0.08 |
|  |  | A-5 | 0.10 |
| BE | B | B-1 | 0.02 |
|  |  | B-2 | 0.04 |
|  |  | B-3 | 0.05 |
|  |  | B-4 | 0.08 |
|  |  | B-5 | 0.10 |
| MDA | M | M-1 | 0.02 |
|  |  | M-2 | 0.04 |
|  |  | M-3 | 0.05 |
|  |  | M-4 | 0.08 |
|  |  | M-5 | 0.10 |

**Table S2** The characteristic peaks and their attributions for 6-AM, BE, and MDA

| Name | Characteristic peaks (cm^-1^) and their adscription |
| --- | --- |
| 6-AM | 1738 (C=O, stretching), 1371 (-CH_3_, bending), 1237 (C-O, stretching), 1055 (C-O, stretching), 1033 (C-O, stretching), 910 (C-H, out-of-plane bending), 840 (C-H, out-of-plane bending) |
| BE | 1715 (C=O, stretching), 1347 (-CH_3_, bending), 1316 (-CH_3_, bending), 1274 (C-N, stretching), 1115 (C-O, stretching), 1071 (C-O, stretching), 1026 (C-O, stretching), 717 (C-H, out-of-plane bending) |
| MDA | 1488 (C=C, stretching), 1441 (C=C, stretching), 1368 (-CH_3_, bending), 1246 (C-O, stretching), 1189 (C-O, stretching), 1039 (C-O, bending), 925 (C-H, out-of-plane bending), and 805 (C-H, out-of-plane bending) |

**Table.S3** PCA analysis parameters using spectral fingerprint regions of urine ATR-FTIR

| Component | R^2^X | R^2^X  (cum) | Eigenvalue | Q^2^ | Q^2^  (cum) | Significance |
| --- | --- | --- | --- | --- | --- | --- |
| 1 | 0.445 | 0.445 | 20 | 0.289 | 0.289 | R1 |
| 2 | 0.206 | 0.651 | 9.27 | 0.328 | 0.522 | R1 |
| 3 | 0.168 | 0.819 | 7.58 | 0.437 | 0.731 | R1 |
| 4 | 0.0848 | 0.904 | 3.82 | 0.436 | 0.848 | R1 |
| 5 | 0.0517 | 0.956 | 2.33 | 0.517 | 0.927 | R1 |
| 6 | 0.0227 | 0.979 | 1.02 | 0.497 | 0.963 | R1 |
| 7 | 0.00756 | 0.986 | 0.34 | 0.298 | 0.974 | R1 |
| 8 | 0.00458 | 0.991 | 0.206 | 0.272 | 0.981 | R1 |

**Table.S4** PCA analysis parameters using full spectral regions of urine ATR-FTIR

| Component | R^2^X | R^2^X  (cum) | Eigenvalue | Q^2^ | Q^2^  (cum) | Significance |
| --- | --- | --- | --- | --- | --- | --- |
| 1 | 0.291 | 0.291 | 13.1 | 0.0247 | 0.0247 | R1 |
| 2 | 0.257 | 0.548 | 11.6 | 0.333 | 0.35 | R1 |
| 3 | 0.189 | 0.736 | 8.49 | 0.391 | 0.604 | R1 |
| 4 | 0.0993 | 0.836 | 4.47 | 0.33 | 0.734 | R1 |
| 5 | 0.0817 | 0.917 | 3.67 | 0.46 | 0.857 | R1 |
| 6 | 0.0403 | 0.958 | 1.81 | 0.457 | 0.922 | R1 |
| 7 | 0.0142 | 0.972 | 0.637 | 0.292 | 0.945 | R1 |
| 8 | 0.00868 | 0.98 | 0.391 | 0.269 | 0.96 | R1 |

**Table.S5** PLS-DA model parameters using spectral fingerprint regions of urine ATR-FTIR spectra in calibration set

| Component | R^2^X | R^2^X  (cum) | Eigenvalue | Q^2^ | Q^2^  (cum) | Significance |
| --- | --- | --- | --- | --- | --- | --- |
| 1 | 0.265 | 0.265 | 7.95 | 0.328 | 0.328 | R1 |
| 2 | 0.194 | 0.459 | 5.81 | 0.245 | 0.493 | R1 |
| 3 | 0.346 | 0.805 | 10.4 | 0.193 | 0.591 | R1 |
| 4 | 0.0918 | 0.897 | 2.75 | 0.262 | 0.698 | R1 |
| 5 | 0.0602 | 0.957 | 1.81 | 0.0762 | 0.721 | R1 |
| 6 | 0.00739 | 0.964 | 0.222 | 0.345 | 0.817 | R1 |
| 7 | 0.0203 | 0.984 | 0.609 | 0.0434 | 0.825 | N4 |

**Table.S6** PLS-DA model parameters using full spectral regions of urine ATR-FTIR spectra in calibration set

| Component | R^2^X | R^2^X  (cum) | Eigenvalue | Q^2^ | Q^2^  (cum) | Significance |
| --- | --- | --- | --- | --- | --- | --- |
| 1 | 0.231 | 0.231 | 6.92 | 0.159 | 0.159 | R1 |
| 2 | 0.152 | 0.383 | 4.57 | 0.377 | 0.476 | R1 |
| 3 | 0.146 | 0.528 | 4.37 | -0.00834 | 0.472 | NS |
| 4 | 0.199 | 0.727 | 5.96 | 0.241 | 0.599 | R1 |
| 5 | 0.149 | 0.876 | 4.46 | 0.313 | 0.725 | R1 |
| 6 | 0.0549 | 0.931 | 1.65 | 0.0268 | 0.732 | NS |
| 7 | 0.0388 | 0.97 | 1.16 | 0.18 | 0.78 | R1 |
| 8 | 0.00747 | 0.977 | 0.224 | 0.0000392 | 0.78 | NS |

**Table.S7** OPLS-DA model parameters using spectral fingerprint regions of urine ATR-FTIR spectra in calibration set

| Component | R^2^X | R^2^X  (cum) | Eigenvalue | Q^2^ | Q^2^  (cum) | Significance |
| --- | --- | --- | --- | --- | --- | --- |
| Model |  | 0.964 |  |  | 0.853 |  |
| Predictive |  | 0.257 |  |  | 0.853 |  |
| P1 | 0.159 | 0.159 | 4.78 | 0.42 | 0.42 | R1 |
| P2 | 0.0975 | 0.257 | 2.92 | 0.433 | 0.853 | R1 |
| Orthogonal in X(OPLS) |  | 0.707 |  |  |  |  |
| O1 | 0.444 | 0.444 | 13.3 |  |  | R1 |
| O2 | 0.165 | 0.61 | 4.95 |  |  | R1 |
| O3 | 0.0686 | 0.678 | 2.06 |  |  | R1 |
| O4 | 0.029 | 0.707 | 0.87 |  |  | R1 |

**Table.S8** OPLS-DA model parameters using full spectral regions of urine ATR-FTIR spectra in calibration set

| Component | R^2^X | R^2^X  (cum) | Eigenvalue | Q^2^ | Q^2^  (cum) | Significance |
| --- | --- | --- | --- | --- | --- | --- |
| Model |  | 0.986 |  |  | 0.883 |  |
| Predictive |  | 0.186 |  |  | 0.883 |  |
| P1 | 0.114 | 0.114 | 3.41 | 0.42 | 0.42 | R1 |
| P2 | 0.0727 | 0.186 | 2.18 | 0.463 | 0.883 | R1 |
| Orthogonal in X(OPLS) |  | 0.799 |  |  |  |  |
| O1 | 0.213 | 0.213 | 6.4 |  |  | R1 |
| O2 | 0.208 | 0.422 | 6.25 |  |  | R1 |
| O3 | 0.246 | 0.668 | 7.38 |  |  | R1 |
| O4 | 0.069 | 0.737 | 2.07 |  |  | R1 |
| O5 | 0.0374 | 0.774 | 1.12 |  |  | R1 |
| O6 | 0.0133 | 0.787 | 0.4 |  |  | R1 |
| O7 | 0.0119 | 0.799 | 0.356 |  |  | R1 |

Stretching vibration refers to the periodic movement of atoms along the direction of chemical bonds, as exemplified by the C=O stretch of acetone at 1716 cm^-1^ (characteristic of carbonyl groups). Bending vibration refers to the periodic movement of atoms perpendicular to the bond axis direction, as demonstrated by the C-H in-plane bending mode of benzene ring at 1036 cm^-1^.


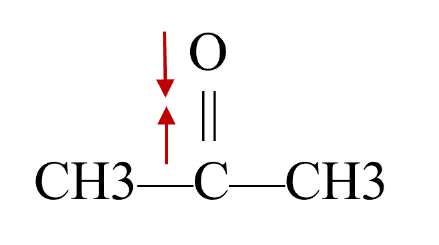

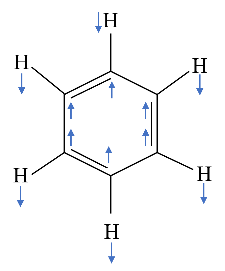


**b**

**a**

**Fig.S1** Characteristic infrared vibrational modes (a) Acetone. (b) Benzene ring.

*The arrows are indicated as the direction of vibrational displacement direction of the atom.


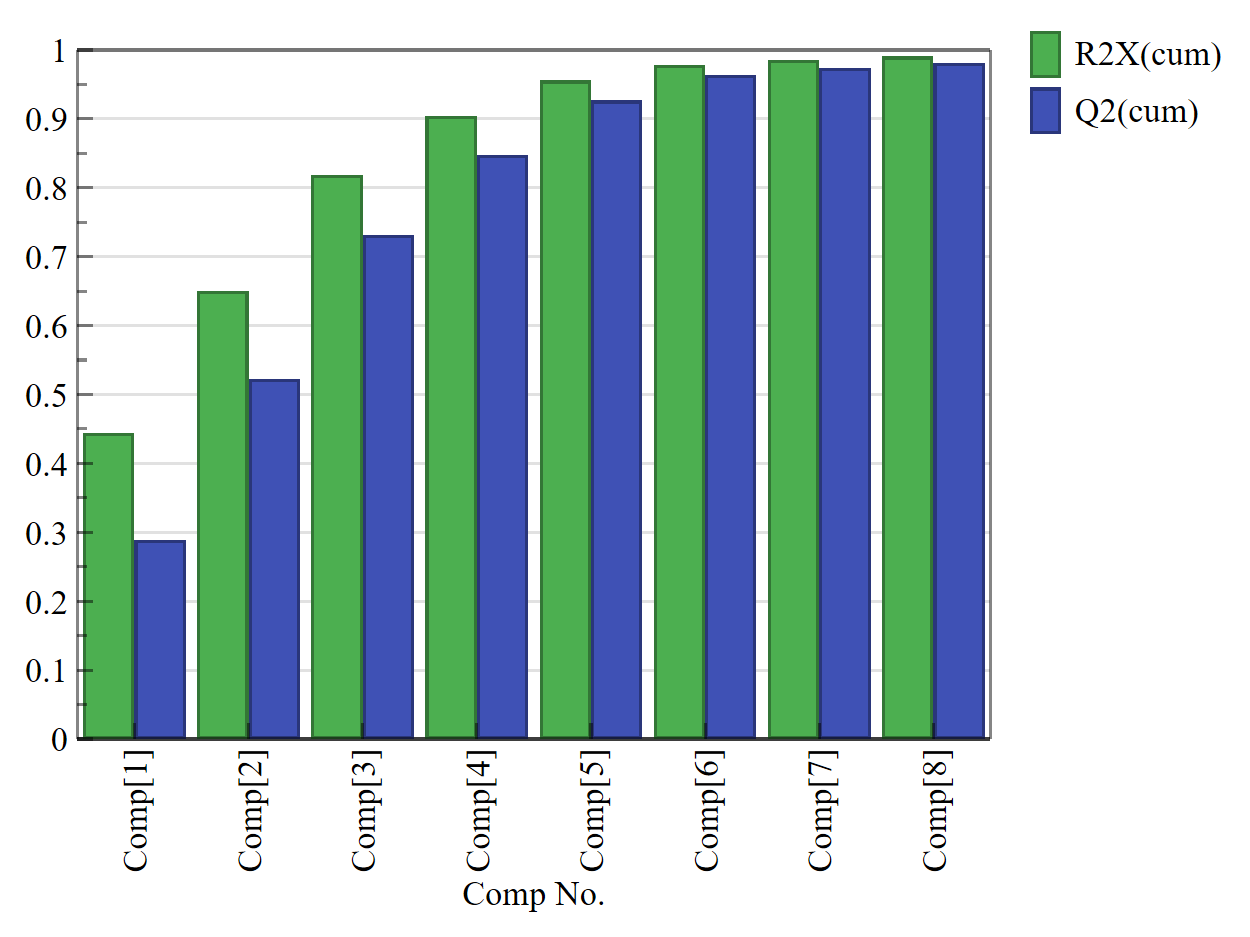

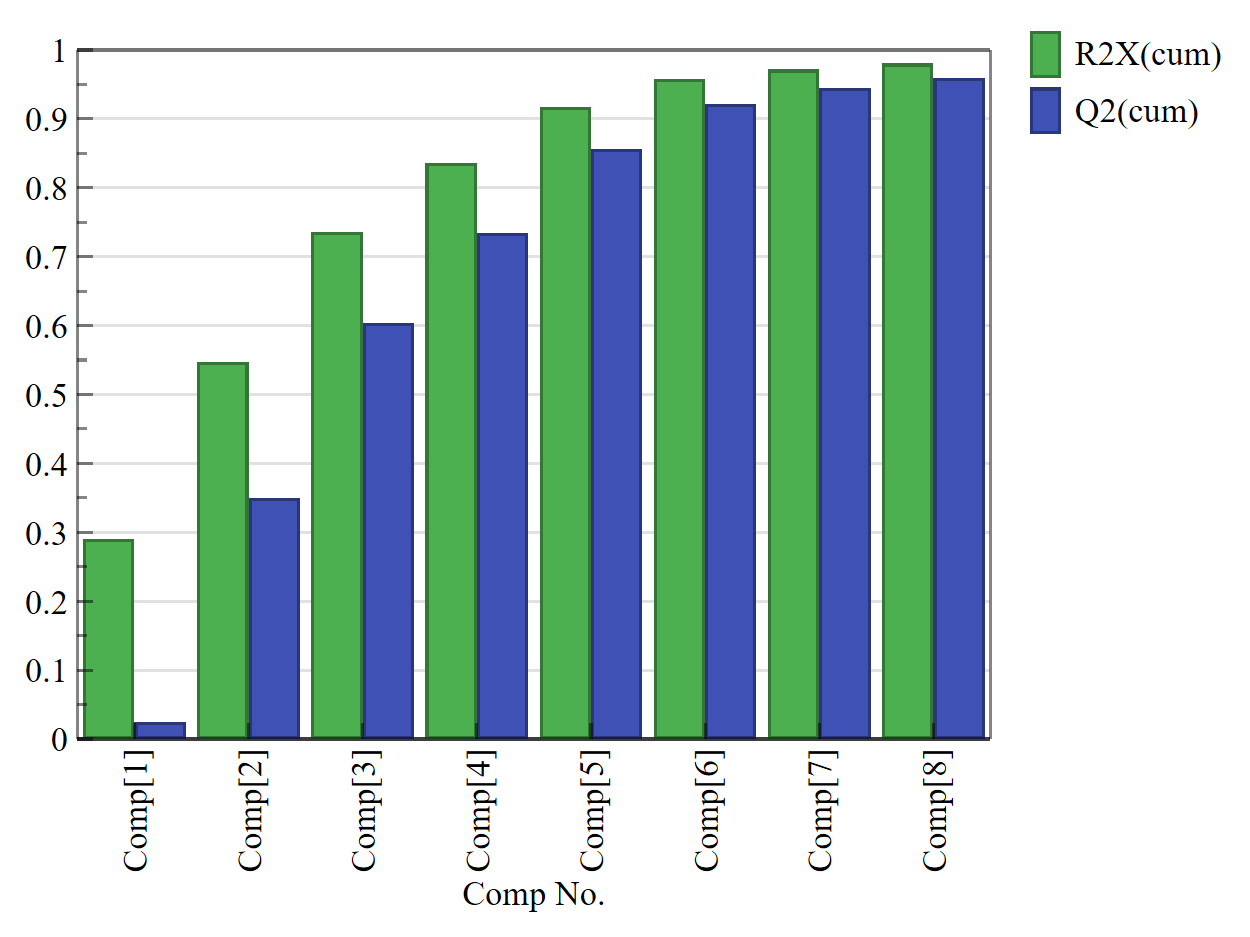
.

**b**

**a**

**Fig.S2** (a)Optimization of PC number of PCA analysis using fingerprint regions of urine ATR-FTIR. (b)Optimization of PC number of PCA analysis using full spectral regions of urine ATR-FTIR.


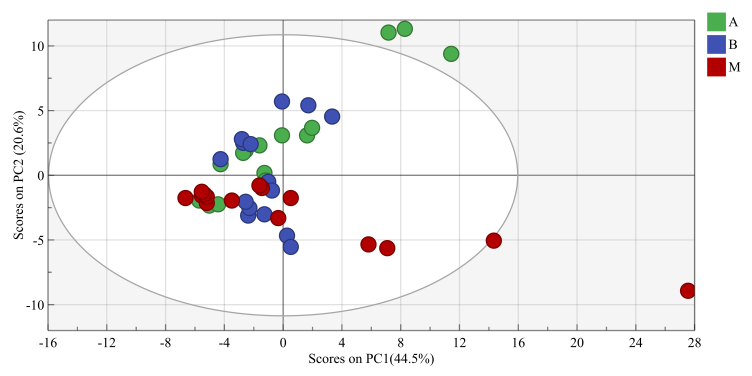

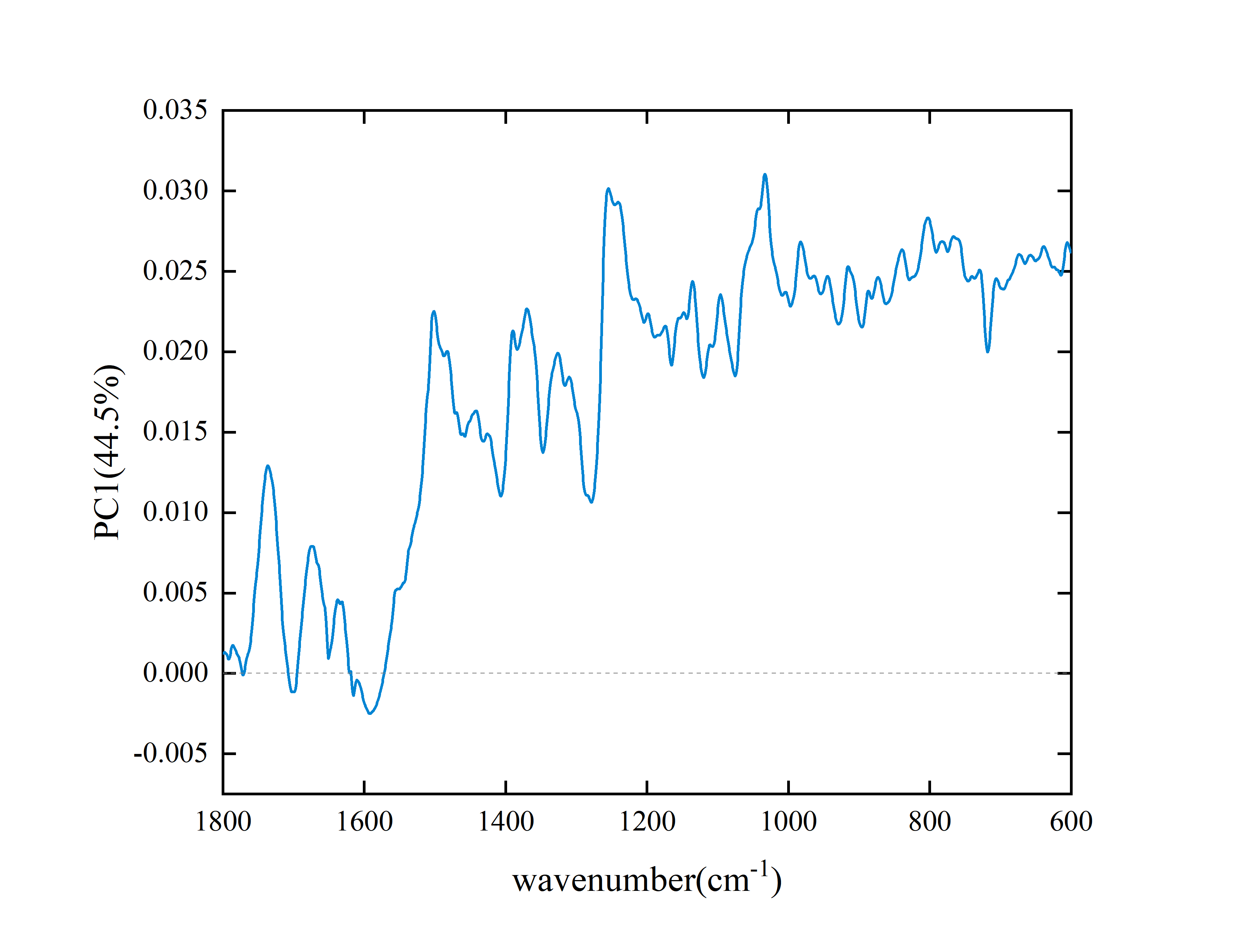


**a**

**b**


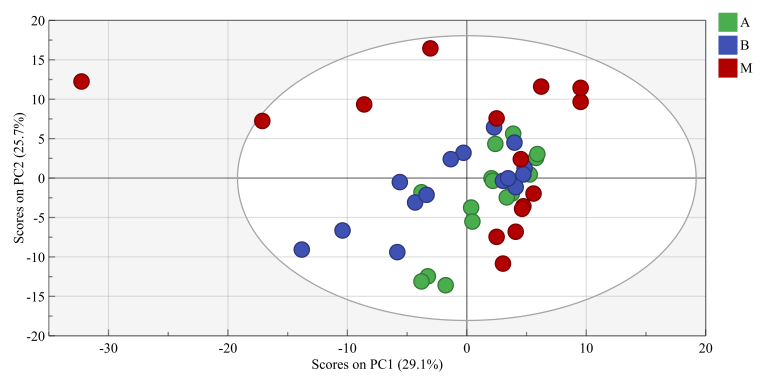

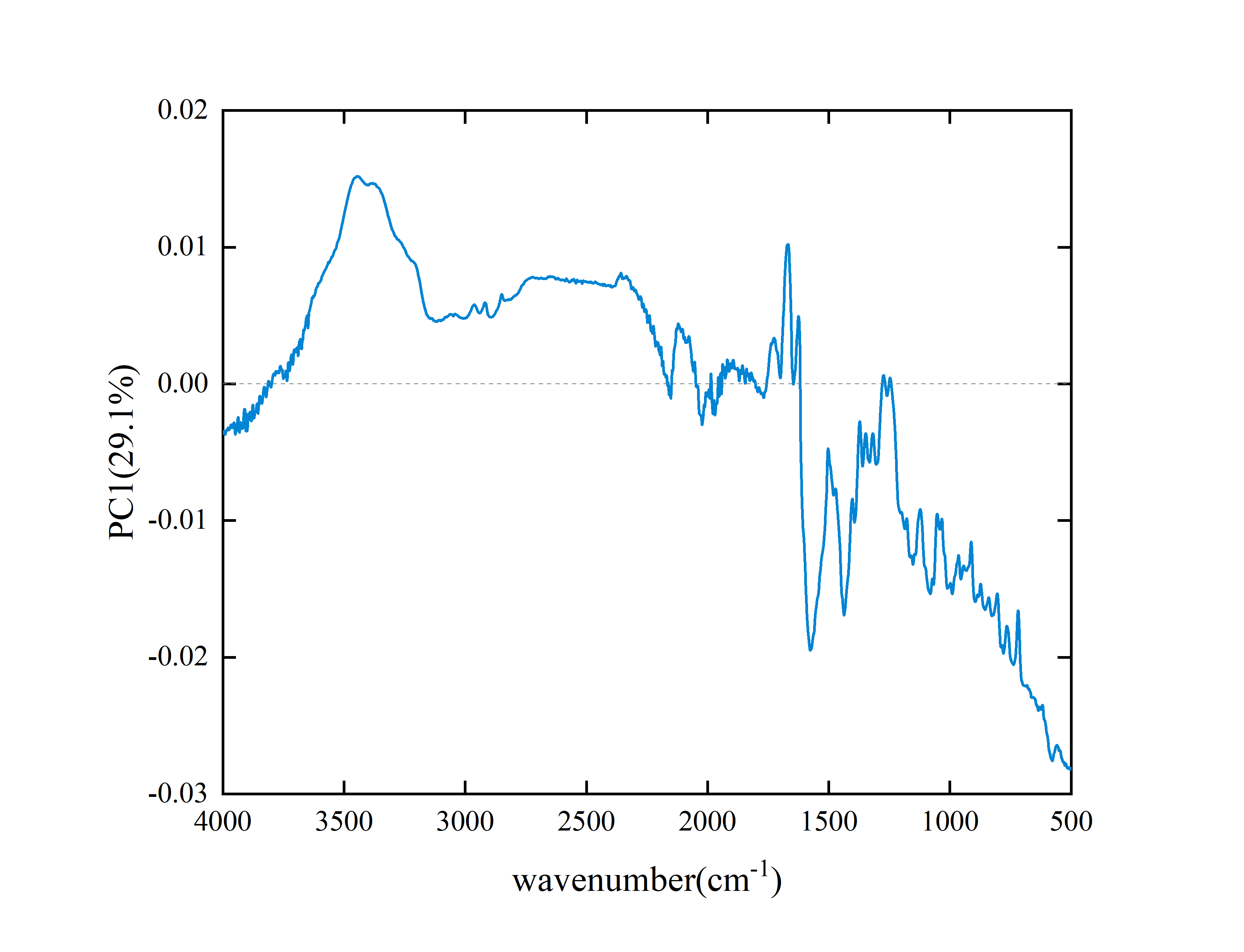


**c**

**d**

**Fig.S3** PCA score plots and loading plots (a)PCA score plot using fingerprint regions. (b)Loading plot of PCA using fingerprint regions. (c)PCA score plot using full spectral regions. (d)Loading plot of PCA using full spectral regions


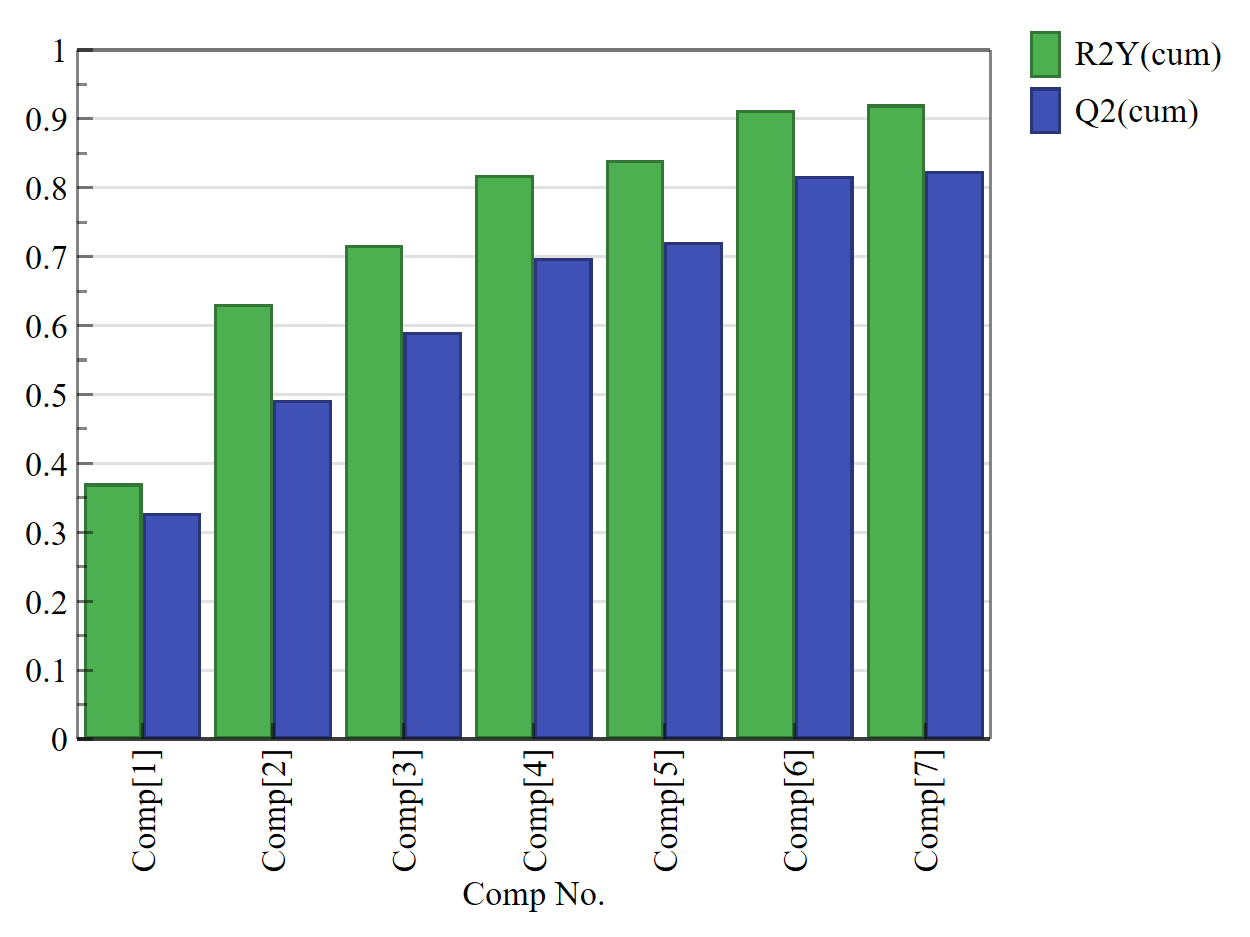

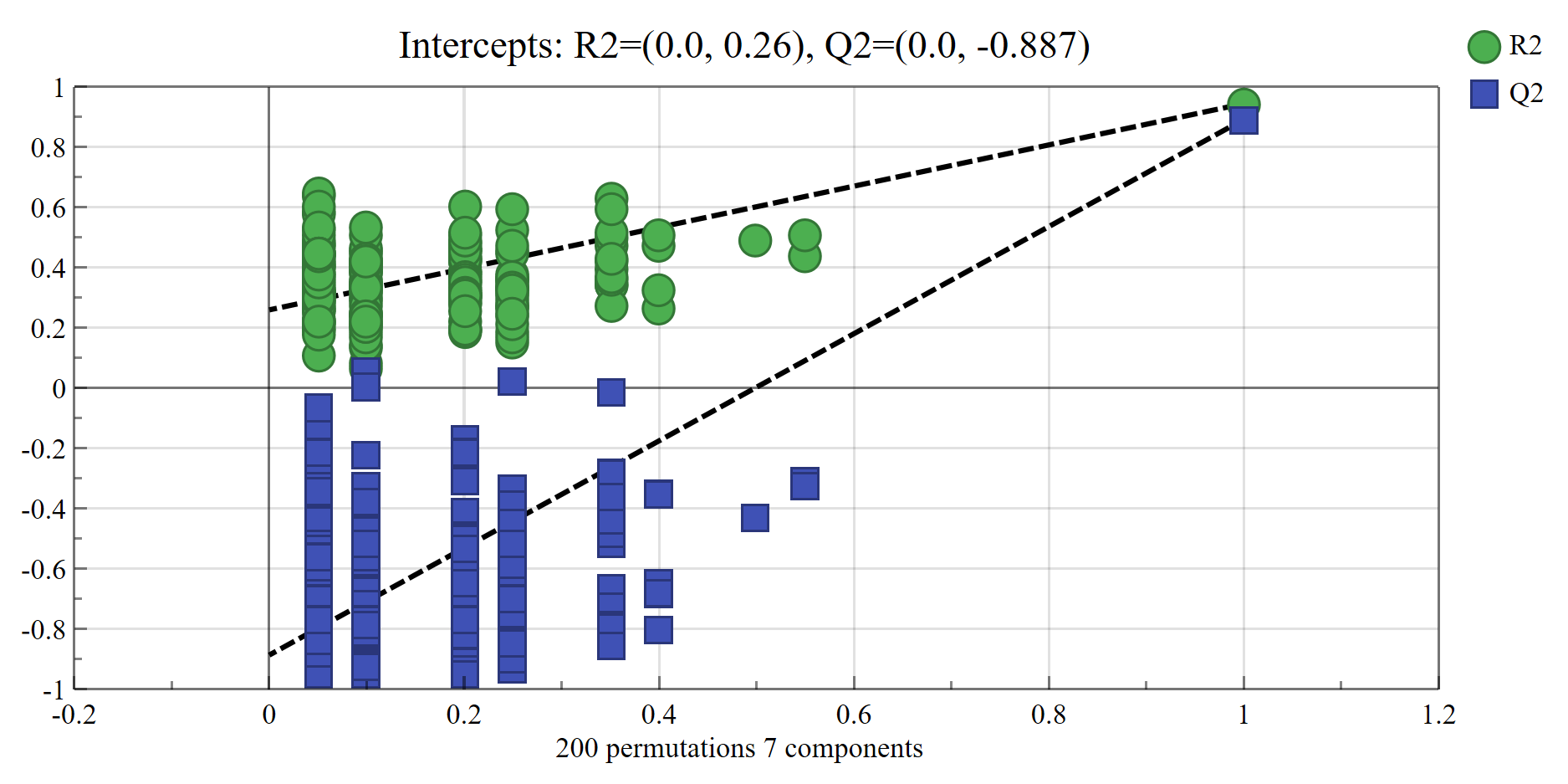


**b**

**a**


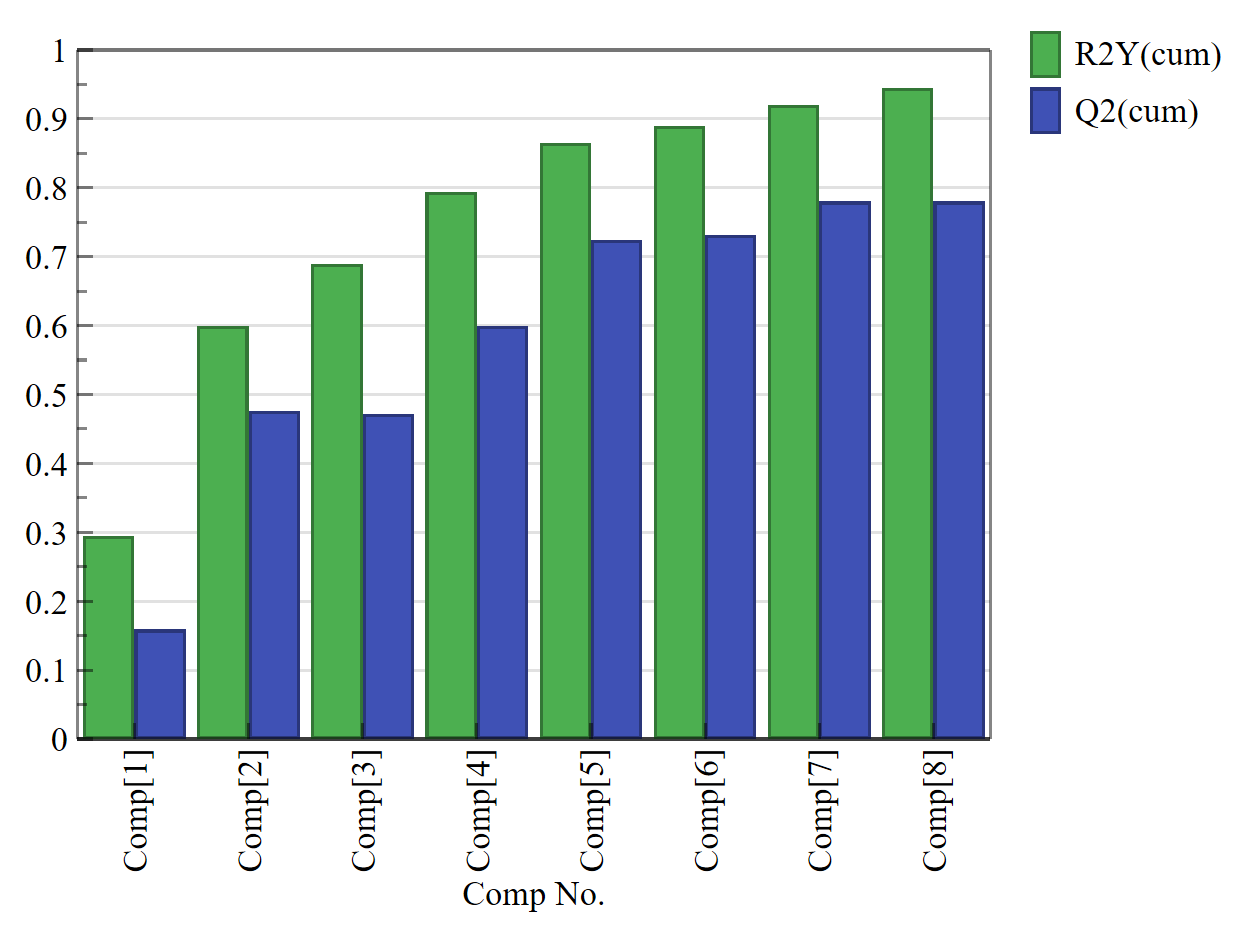

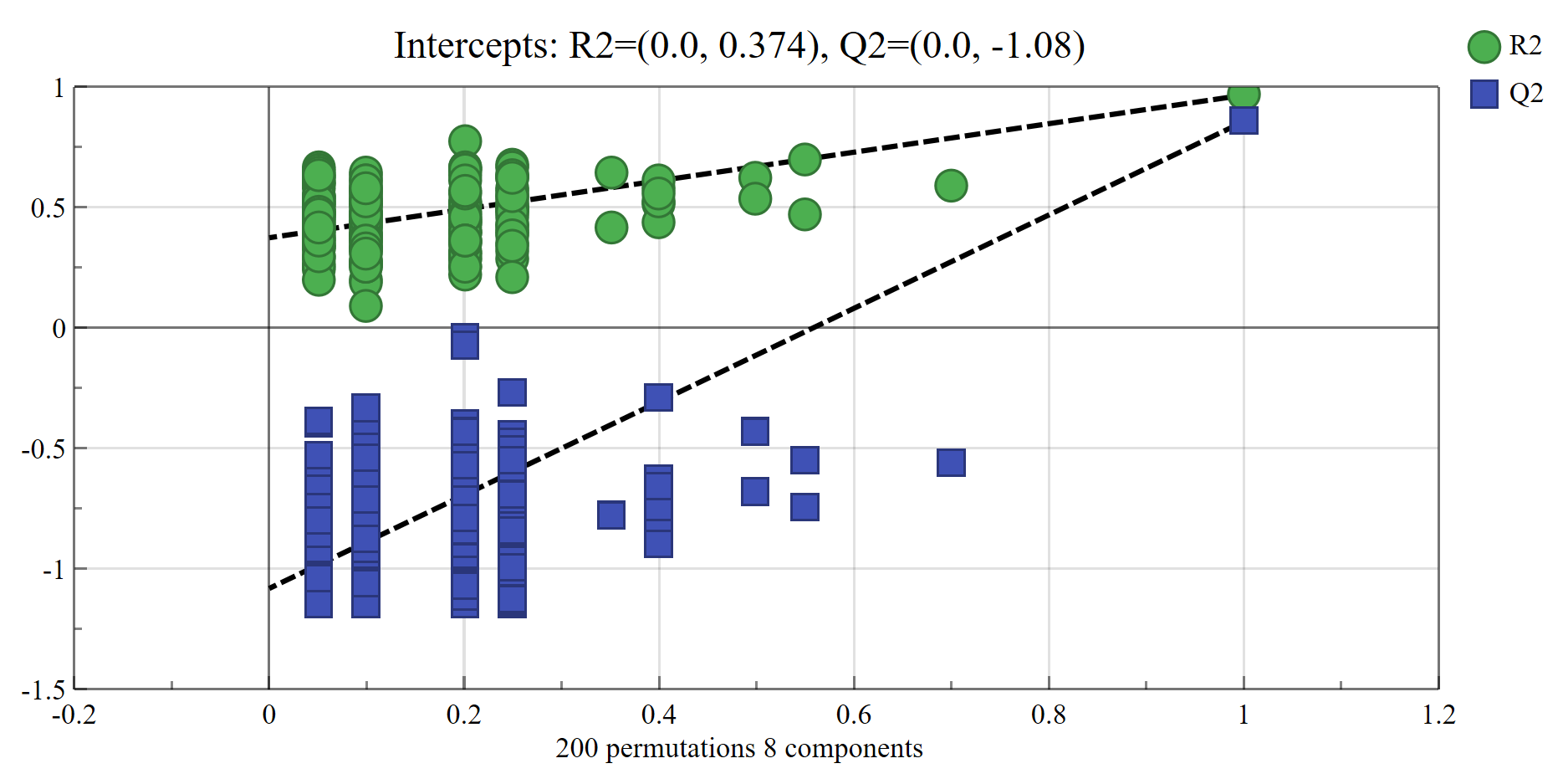


**d**

**c**

**Fig.S4** PLS-DA model PC optimization and corresponding R^2^ and Q^2^ (a)Optimization of PC number of PLS-DA model using fingerprint regions of urine ATR-FTIR and corresponding R^2^ and Q^2^. (b)Permutation of PLS-DA model using fingerprint regions of urine ATR-FTIR. (c)Optimization of PC number of PLS-DA model using full spectral regions of urine ATR-FTIR and corresponding R^2^ and Q^2^. (d)Permutation of PLS-DA model using full spectral regions of urine ATR-FTIR.


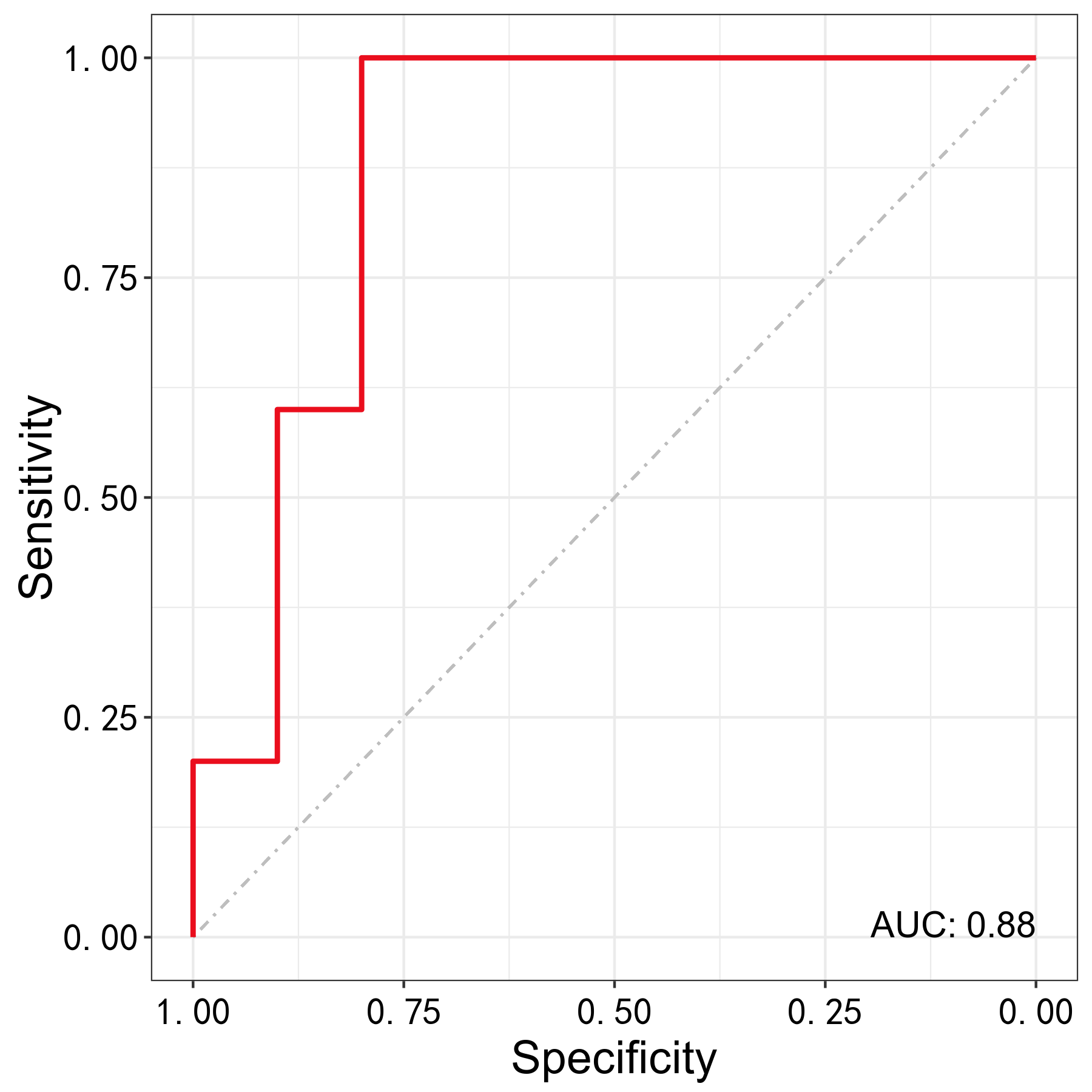

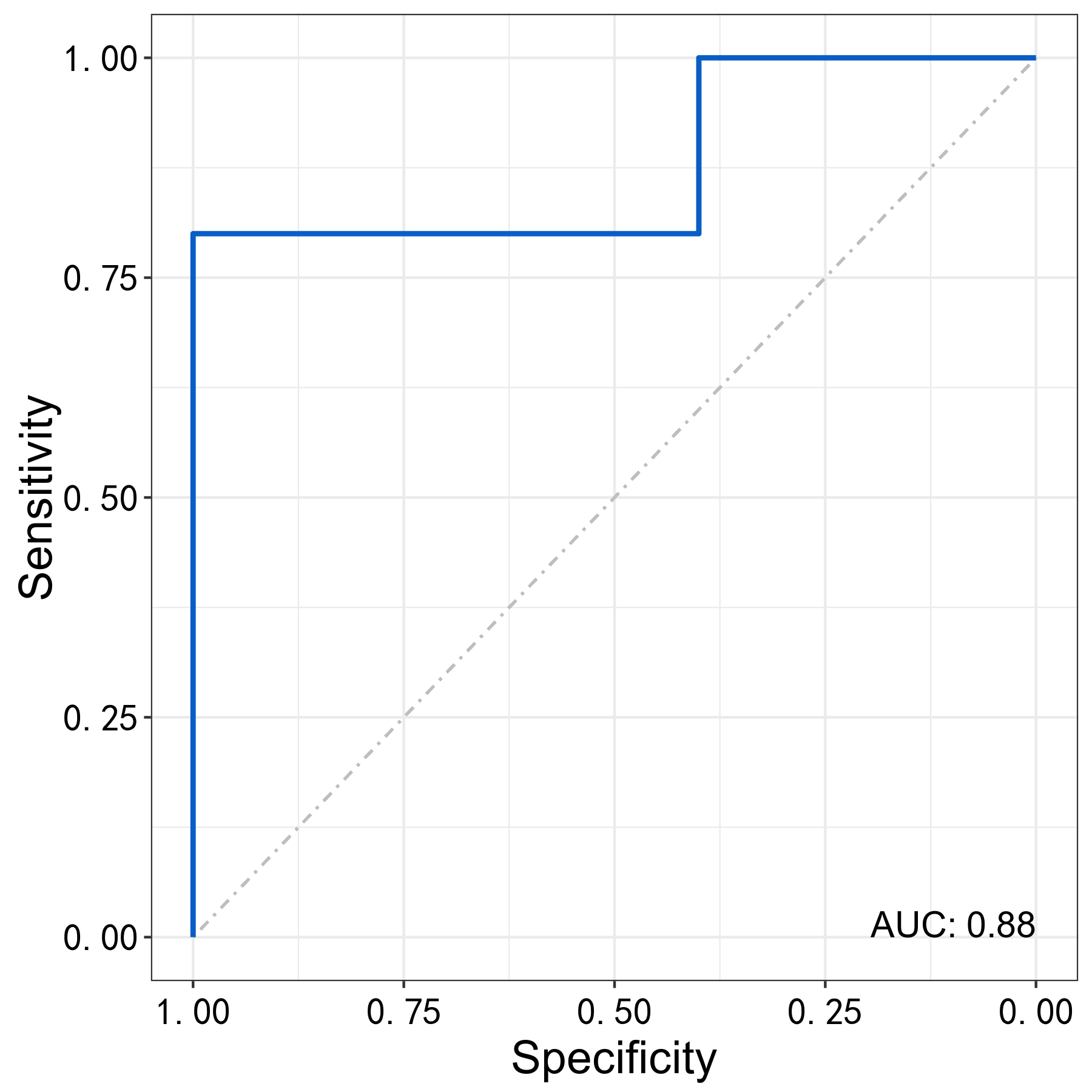

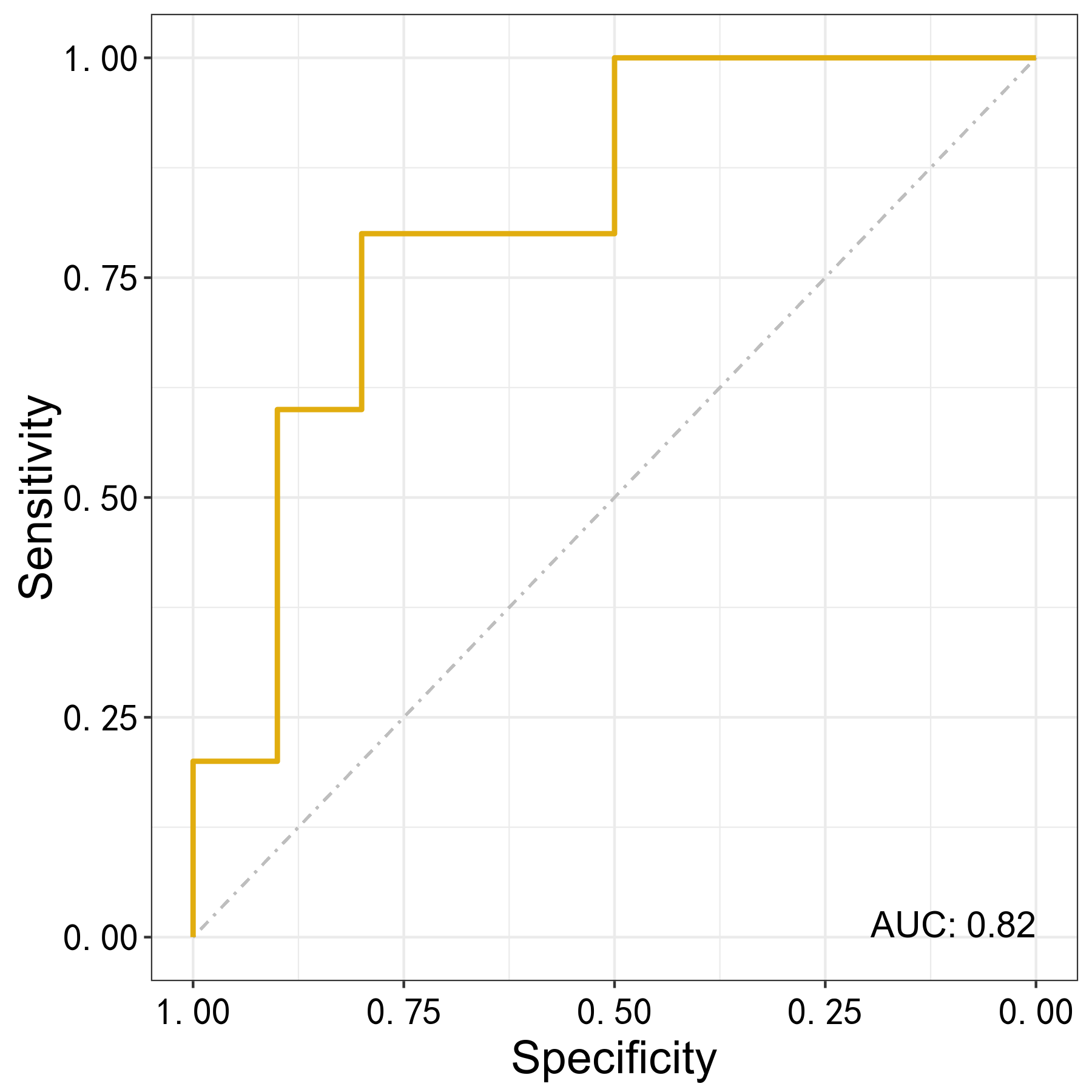


**b**

**a**

**c**

**Fig.S5** ROC analysis of PLS-DA model using fingerprint regions of urine ATR-FTIR (a) Experimental group: Group A; Control group: Group B and M. (b) Experimental group: Group B; Control group: Group A and M. (c) Experimental group: Group M; Control group: Group A and B.

^
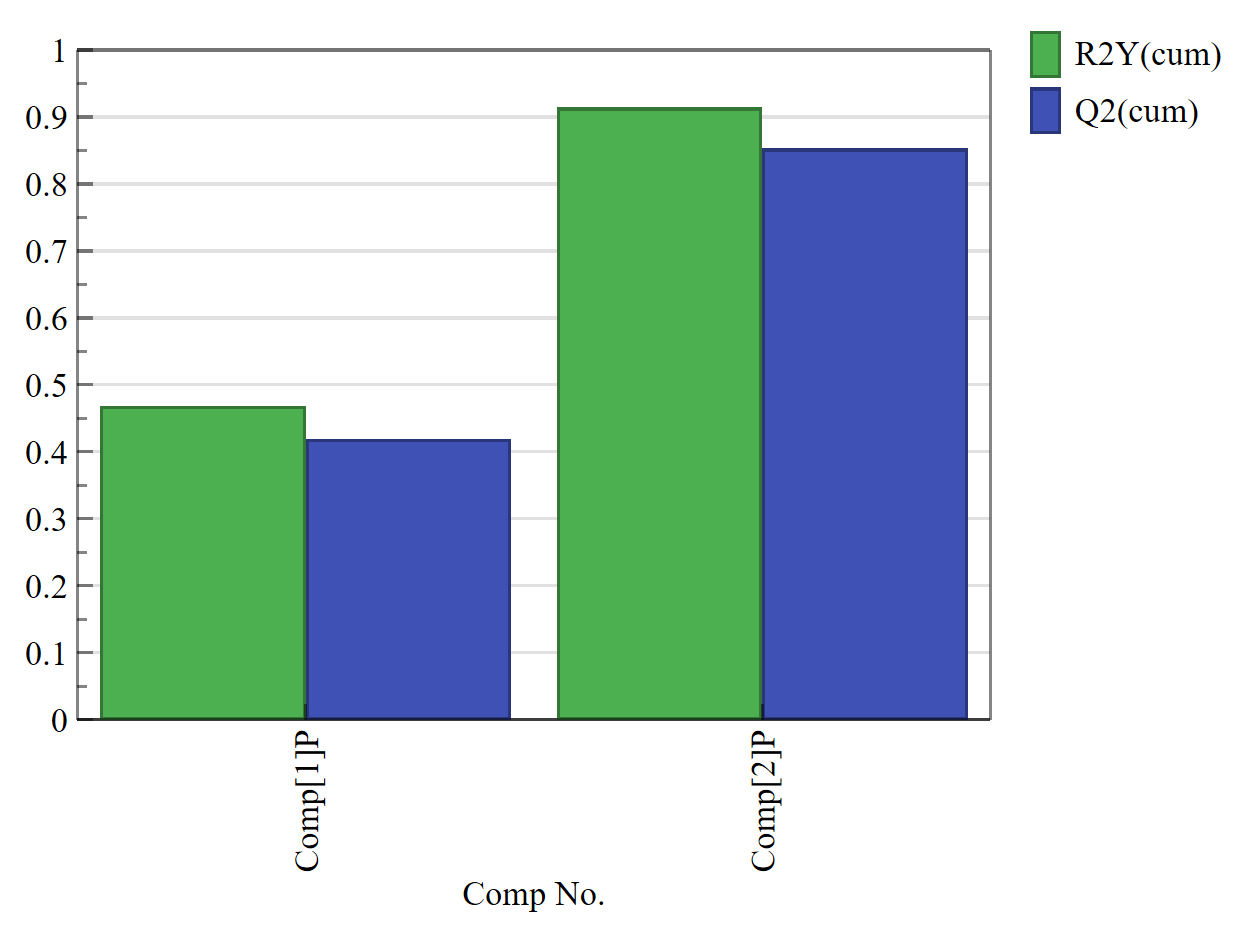

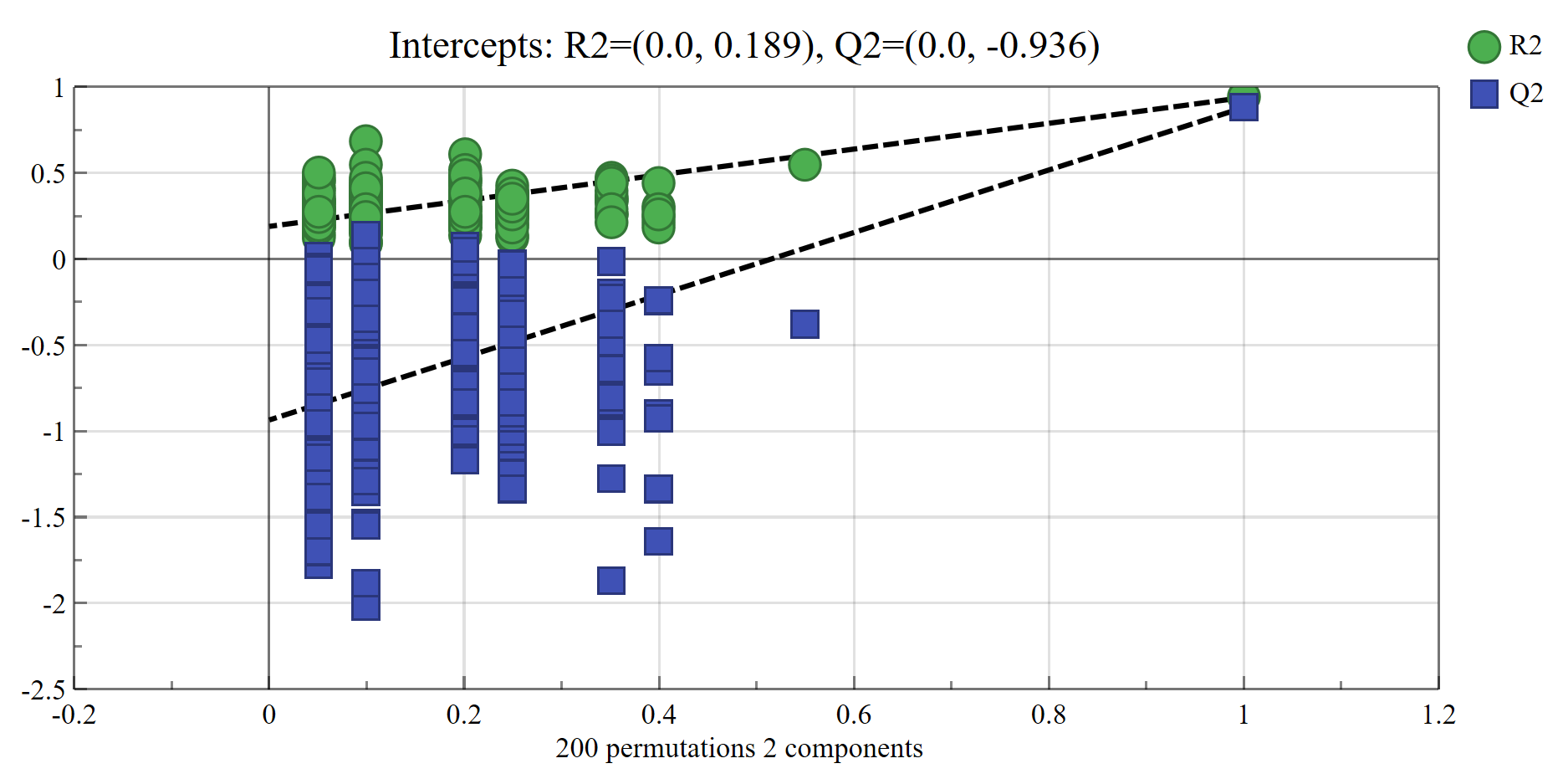
^

**b**

**a**

^
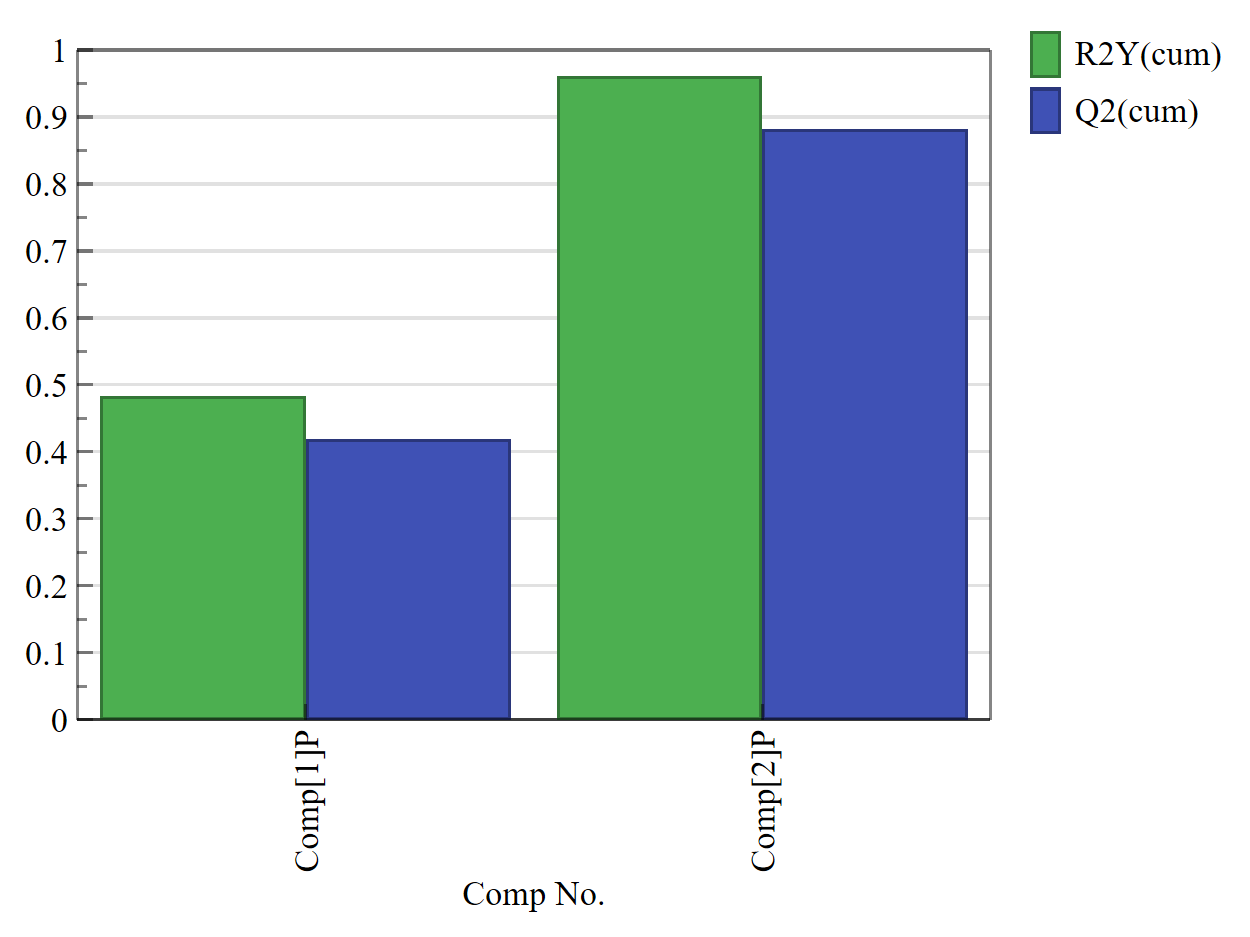

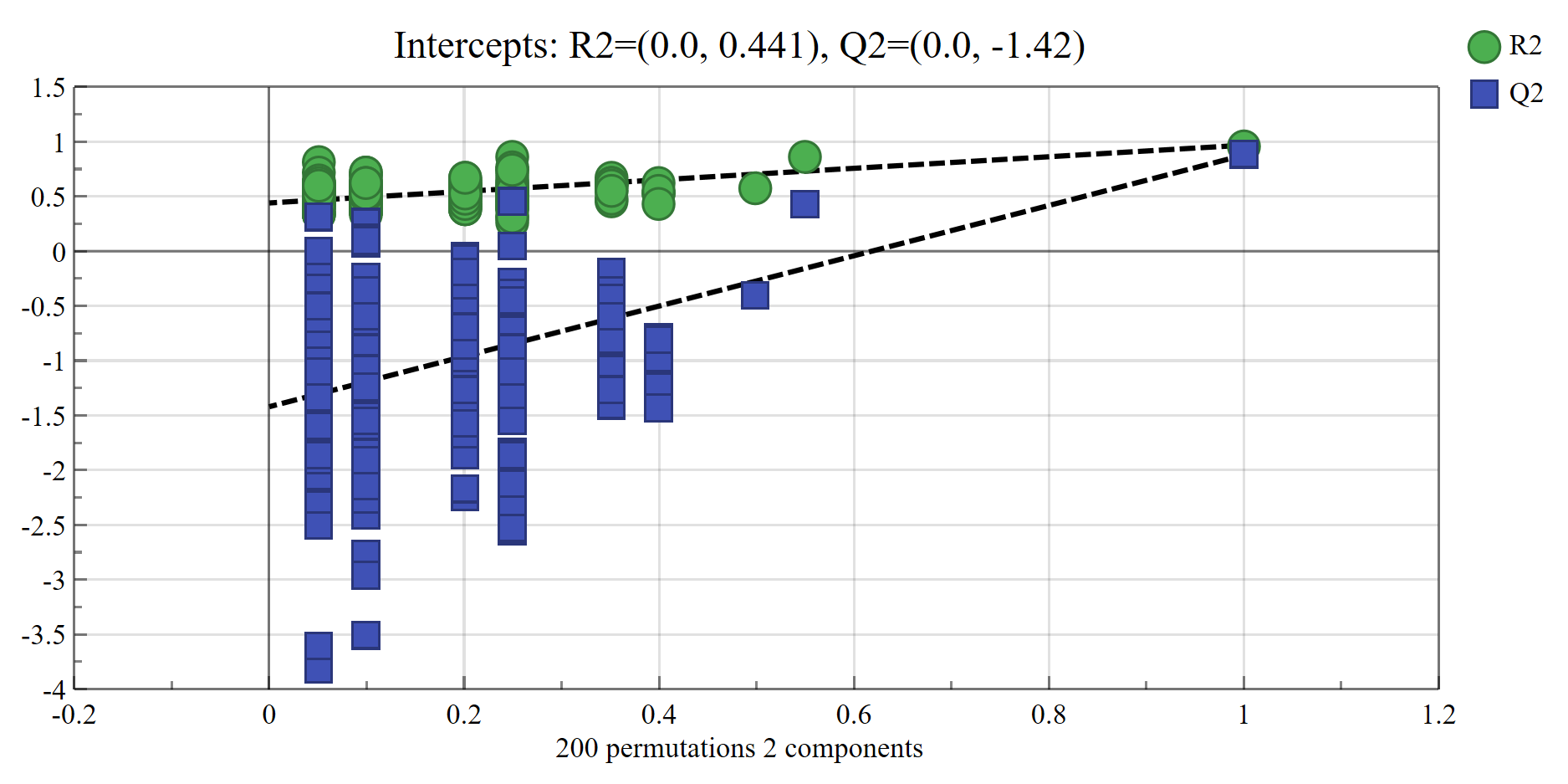
^

**d**

**c**

**Fig.S6** OPLS-DA model PC optimization and corresponding R^2^ and Q^2^ (a)Optimization of PC number of OPLS-DA model using fingerprint regions of urine ATR-FTIR and corresponding R^2^ and Q^2^. (b)Permutation of OPLS-DA model using fingerprint regions of urine ATR-FTIR. (c)Optimization of PC number of OPLS-DA model using full spectral regions of urine ATR-FTIR and corresponding R^2^ and Q^2^. (d)Permutation of OPLS-DA model using full spectral regions of urine ATR-FTIR.

^
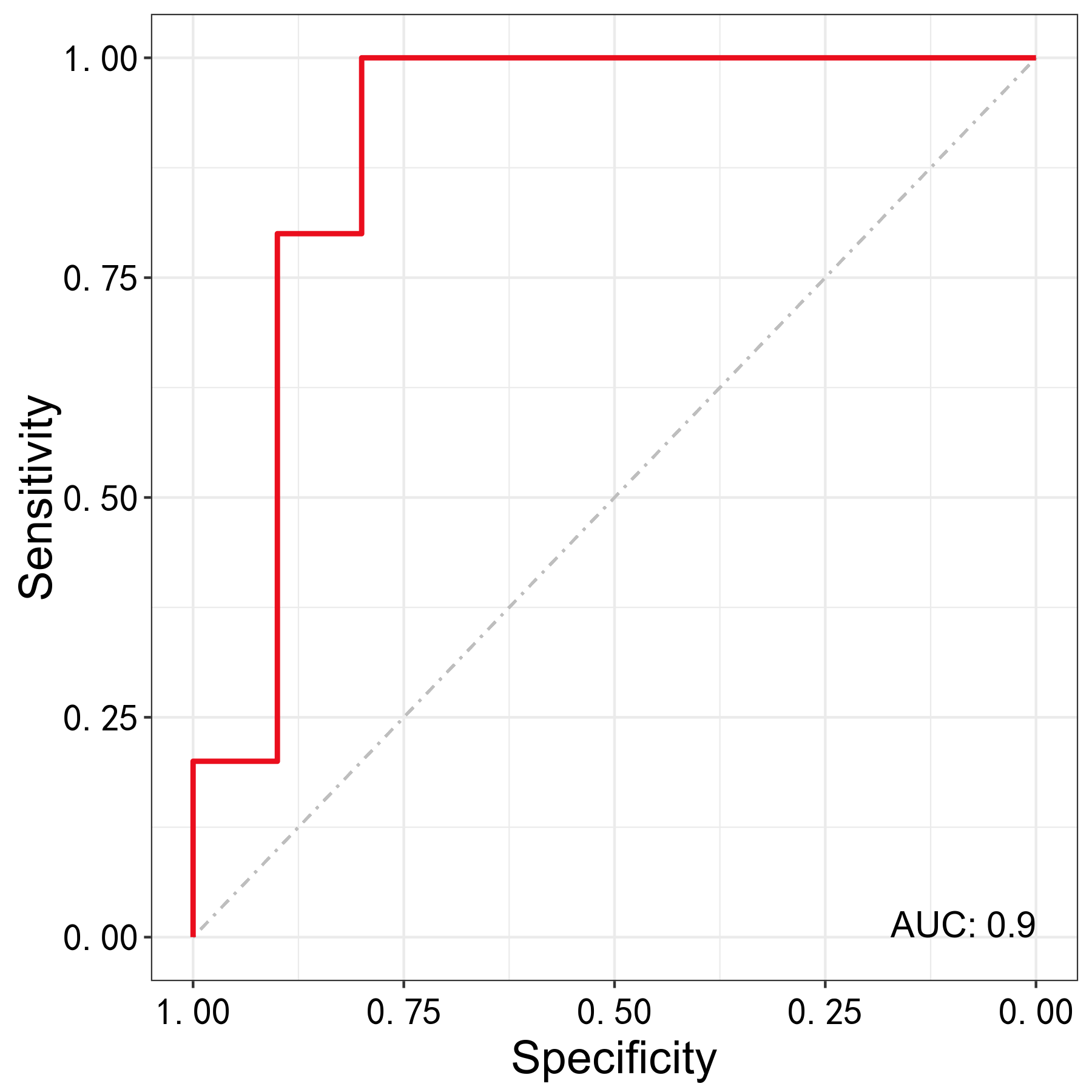

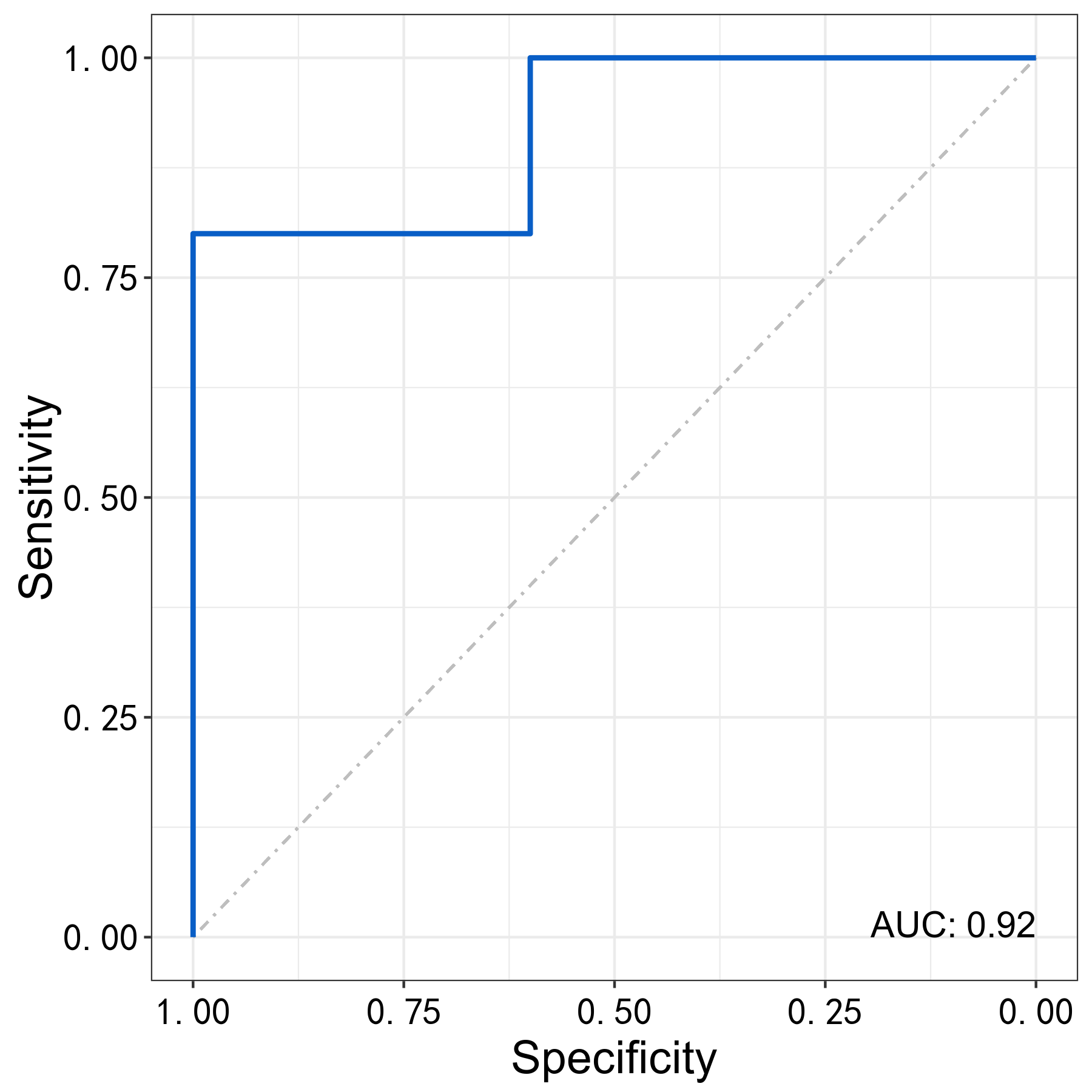

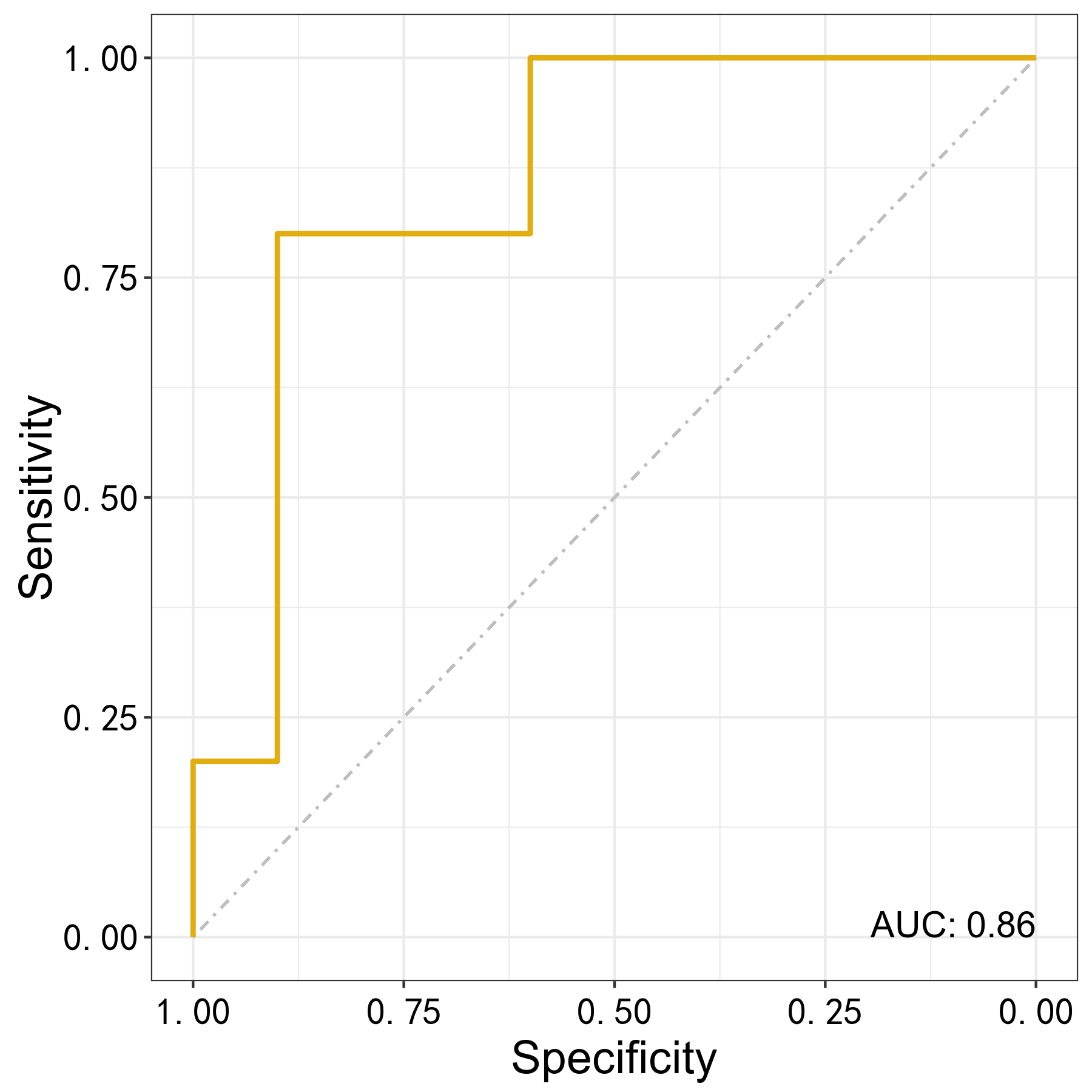
^

**b**

**c**

**a**

**Fig.S7** ROC analysis of OPLS-DA model using fingerprint regions of urine ATR-FTIR (a) Experimental group: Group A; Control group: Group B and M. (b) Experimental group: Group B; Control group: Group A and M. (c) Experimental group: Group M; Control group: Group A and B.

^
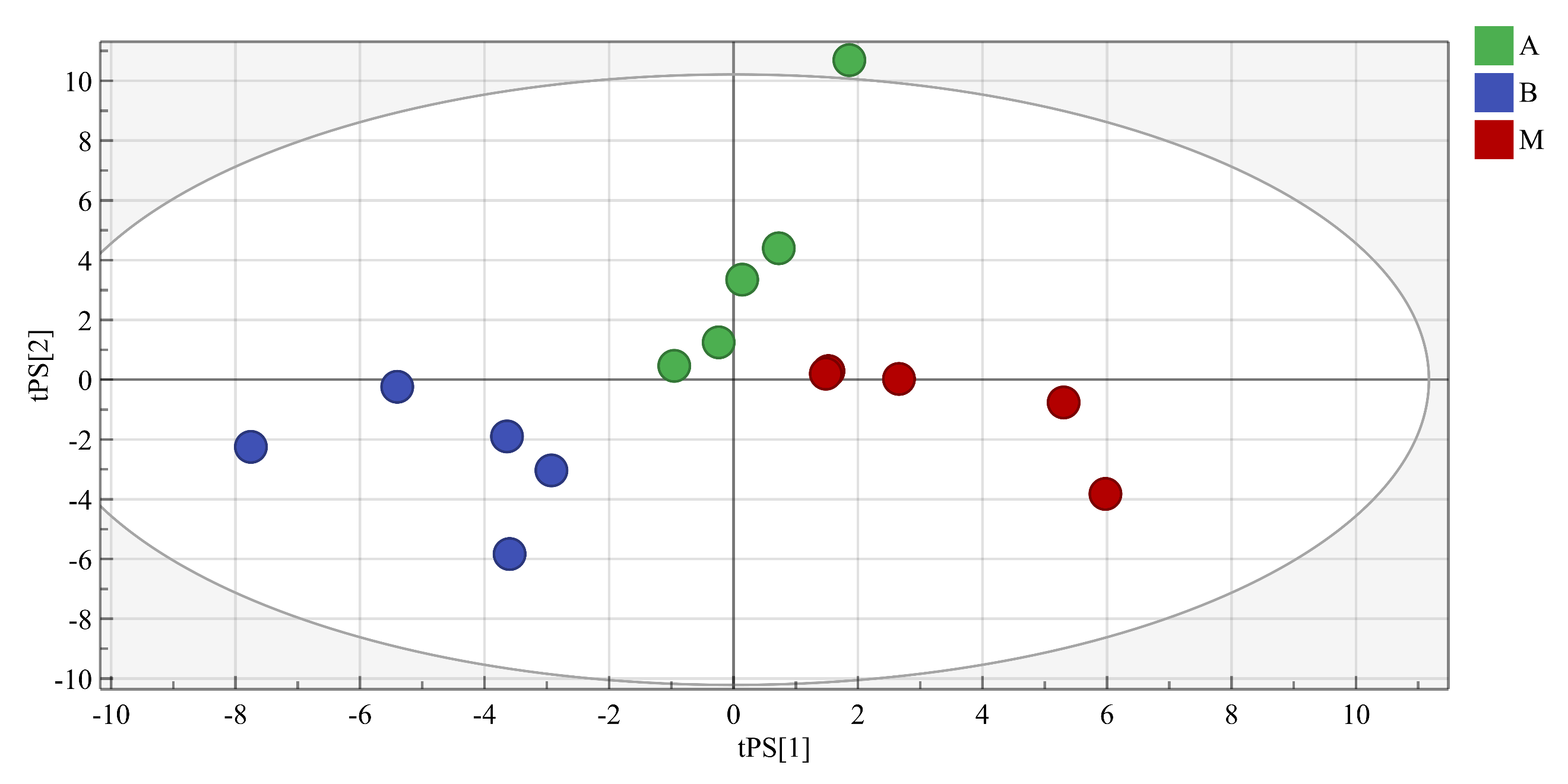

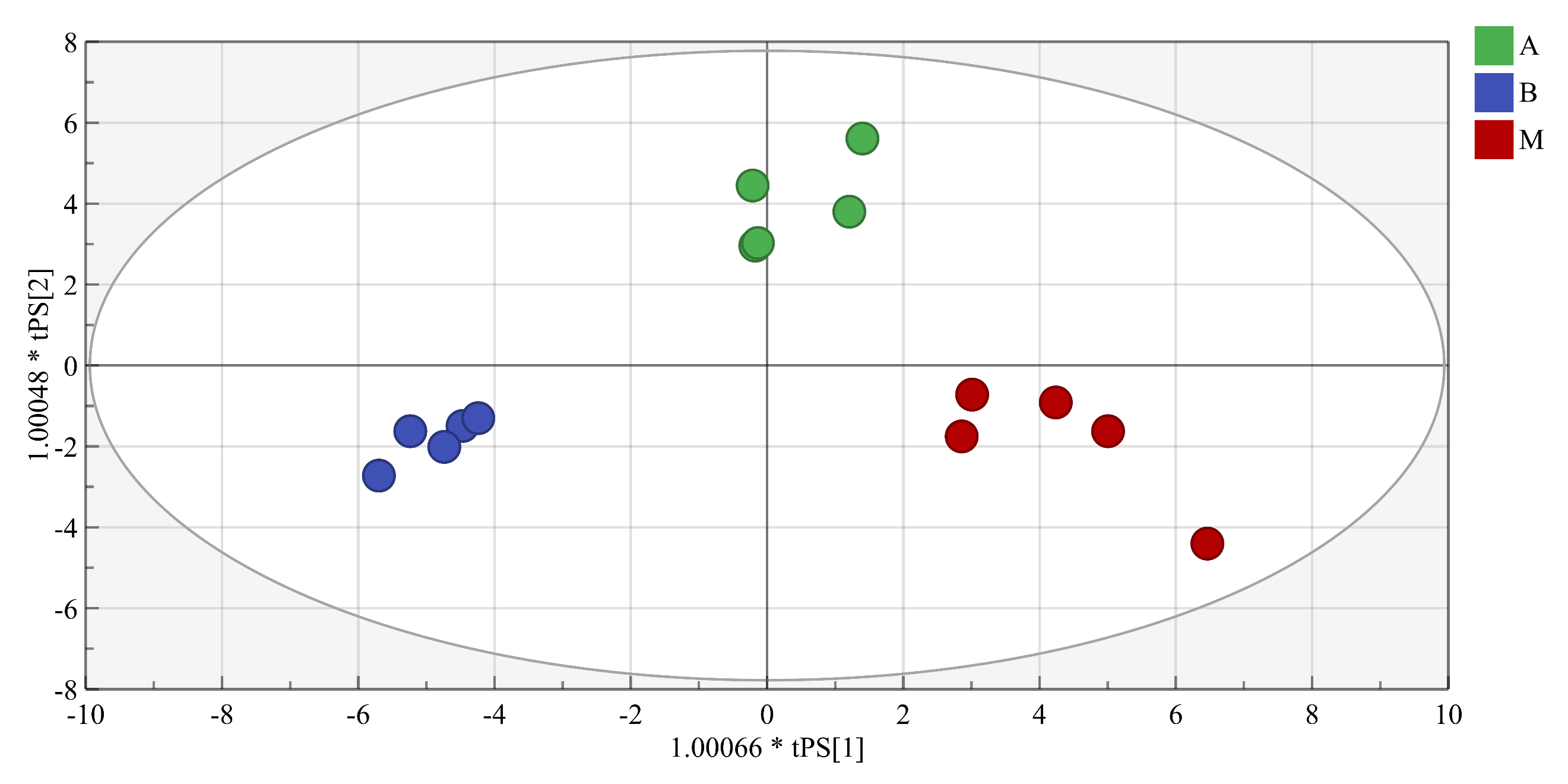
^

**a**

**b**

**Fig.S8** PLS-DA and OPLS-DA score plots (a) PLS-DA model score plot using fingerprint regions in an independent validation set. (b) OPLS-DA model score plot using fingerprint regions in an independent validation set.
